# Transcriptional Homeostasis of Oxidative Stress-Related Pathways in Altered Gravity

**DOI:** 10.3390/ijms19092814

**Published:** 2018-09-18

**Authors:** Svantje Tauber, Swantje Christoffel, Cora Sandra Thiel, Oliver Ullrich

**Affiliations:** 1 Institute of Anatomy, Faculty of Medicine, University of Zurich, Winterthurerstrasse 190, 8057 Zurich, Switzerland; svantje.tauber@uzh.ch (S.T.); swantje.christoffel@uzh.ch (S.C.); 2 Department of Machine Design, Engineering Design and Product Development, Institute of Mechanical Engineering, Otto-von-Guericke-University Magdeburg, Universitätsplatz 2, 39106 Magdeburg, Germany; 3 Space Life Sciences Laboratory (SLSL), Kennedy Space Center, 505 Odyssey Way, Exploration Park, FL 32953, USA; 4 Zurich Center for Integrative Human Physiology (ZIHP), University of Zurich, Winterthurerstrasse 190, 8057 Zurich, Switzerland

**Keywords:** innate immunity, adaptive immunity, spaceflight, microgravity, gravisensitivity, microarray

## Abstract

Whereby several types of cultured cells are sensitive to gravity, the immune system belongs to the most affected systems during spaceflight. Since reactive oxygen species/reactive nitrogen species (ROS/RNS) are serving as signals of cellular homeostasis, particularly in the cells of the immune system, we investigated the immediate effect of altered gravity on the transcription of 86 genes involved in reactive oxygen species metabolism, antioxidative systems, and cellular response to oxidative stress, using parabolic flight and suborbital ballistic rocket experiments and microarray analysis. In human myelomonocytic U937 cells, we detected a rapid response of 19.8% of all of the investigated oxidative stress-related transcripts to 1.8 g of hypergravity and 1.1% to microgravity as early as after 20 s. Nearly all (97.2%) of the initially altered transcripts adapted after 75 s of hypergravity (max. 13.5 g), and 100% adapted after 5 min of microgravity. After the almost complete adaptation of initially altered transcripts, a significant second pool of differentially expressed transcripts appeared. In contrast, we detected nearly no response of oxidative stress-related transcripts in human Jurkat T cells to altered gravity. In conclusion, we assume a very well-regulated homeostasis and transcriptional stability of oxidative stress-related pathways in altered gravity in cells of the human immune system.

## 1. Introduction

Gravitational force and “oxidative stress” are constant determinants of Earth’s evolutionary history [1,2] and probably played a crucial role in the evolution of life [2,3,4] and its homeostasis [2,5]. Cellular and molecular processes induced and regulated by reactive oxygen and nitrogen species (ROS and RNS), and therefore referred to as “oxidative stress” [6], were first perceived as largely negative and pathological, but are now understood as “physiological states” that elicit a decisive shift in patterns of gene expression [2]. Oxidative stress is considered not as a simple by-product of respiration [7], but rather as an intrinsically driving force of the evolution of life on Earth [2], including chemiosmotic coupling, mitonuclear coadaptation [2], and the evolution of symbiotic associations [4]. ROS/RNS are serving as signals in many diverse circumstances and as a central part of cellular homeostasis [2]. Oxidative stress and damage has been identified as a contributing factor to spaceflight-related dysregulation of the bone [8,9,10,11,12], the cardiovascular system [13], the immune system, and metabolism [14,15]. Thus, normalizing the redox homeostasis could mitigate a portion of the adverse phenomena seen in spaceflight, increasing the level of health and safety during exploration class missions [16].

The release of reactive oxygen species/reactive nitrogen species (ROS/RNS) by macrophages not only represents one of the key elements in the innate immune response as the most important barrier against microbes invading the body [17], redox signals also regulate major histocompatibility complex (MHC) class II-mediated antigen presentation [18], macrophage differentiation [19], pro-inflammatory transcription factors [20,21,22] and transcriptions through chromatin-modulation signals such as histone acetylation and methylation [22]. ROS/RNS control complex signal networks during development, differentiation, and immunomodulation, which are independent of macrophage function during host defense [22,23], and therefore represent key elements in the evolution of cellular functions. Nicotinamide adenine dinucleotide phosphate (NADPH) oxidases, the enzymes which transport electrons and generate reactive oxygen species (ROS) that are responsible for the macrophageal killing of bacteria [24,25], were present from the earliest stages of evolution in every type of multicellular life [26].

All terrestrial life, including humans, has adapted to this fundamental force [27]. Altered gravitational forces result in the strong responses of physiological systems, representing adaptation processes to the new gravitational environment in the time course of hours until weeks [28]. In a vast amount of studies, microgravity has been shown to severely effect and regulate cellular and molecular processes, including morphological changes, proliferation, growth, differentiation, signal transduction, and gene expression (reviewed by Choukèr and Ullrich [29]). Consequently, understanding the adaption processes is crucial for an integrated risk assessment for exploration class space missions [30]. This is in line with the recommendation by the National Academies of Science, Engineering, and Medicine of the United States (US) to investigate the reversibility of the changes that occur during and after flight more carefully [31].

Due to its key role in macrophageal signaling and host defense, we therefore recently investigated the oxidative burst reaction in mammalian macrophages through real-time on orbit measurements during the International Space Station (ISS) experiment TRIPLE LUX A, performed in the BIOLAB laboratory of the ISS COLUMBUS module. We revealed a rapid sensitivity of the oxidative burst reaction to microgravity, followed by a subsequent rapid full adaptation in only 42 s [32]. Due to the rapid adaptation of the oxidative burst to an altered gravitational environment, the question of the adaptivity of oxidative-stress-associated cellular pathways in the cells of the immune system arises.

Not only macrophages are affected by ROS or an altered redox state, but also the activation, proliferation, and differentiation of T cells, thereby modulating their function, particularly at the interface between macrophages as antigen-presenting cells and T cells [33,34]. Therefore, we investigated the transcriptome of the oxidative-stress pathway response in microgravity and hypergravity, using different research platforms, gravity conditions, and time points to identify the potential dysregulation or adaptation of transcripts of genes involved in ROS metabolism, antioxidative systems, and cellular response to oxidative stress. Our approach allowed the identification and validation of gravity-regulated gene expression in oxidative stress pathways through two fully independent large-scale research campaigns, and therefore with a high level of evidence.

## 2. Results

### 2.1. Parabolic Flight and Suborbital Ballistic Rocket Experiments with Human Myelomonocytic U937 Cells and Jurkat T Cells

As already described by Thiel [35,36], human U937 myelomonocytic cells and human Jurkat T cells were subjected to 20 s of hypergravity (1.8 g) and subsequently to 20 s of microgravity during the first parabola of parabolic flight campaigns (19th and 23rd German Aerospace Center (DLR) PFC), and samples were obtained at the end of each flight phase. Control samples were obtained in-flight five min before the first parabola and on ground in the identical hardware and under identical conditions except for the gravitational force (Table 1, Supplementary Figure S1). In case of the TEXUS-49 and TEXUS-51 suborbital ballistic rocket experiments, samples were acquired 75 s after lift-off after the hypergravity launch phase and before the microgravity phase, and after five min of the microgravity phase. Control samples were obtained on ground in the identical hardware and under identical conditions except for the gravitational force. During the TEXUS-51 experiment, additional 1g controls were performed during the microgravity flight phase using an on-board centrifuge, and an additional 1g controls were performed on the ground under regular cell culture conditions to control the hardware effect on the experiment (Table 1, Supplementary Figure S1). For both campaigns, RNA from at least four samples in each group was isolated, labeled, and hybridized on microarray chips (see Materials and Methods).

### 2.2. Analysis of Oxidative Stress-Related Transcripts

The 86 selected oxidative stress-related genes (Supplementary Table S1) were represented by 188 transcripts in the applied NimbleGen arrays based on the hg18 annotation (for U937 cells) and 106 transcript clusters from the Affymetrix GeneChip^®^ Human Transcriptome Array (Thermo Fisher Scientific, Waltham, MA, USA) for Jurkat T cells and screened for differences in gene expression during the respective gravity conditions. We furthermore assigned the 188 investigated transcripts to “anti-oxidant” or “pro-oxidant” according to the classification in the RT² Profiler™ polymerase chain reaction (PCR) Array Human Oxidative Stress (Qiagen, Hilden, Germany) and National Center for Biotechnology Information (NCBI) database. The investigated 188 transcripts comprise 115 (61%) anti-oxidant transcripts, 19 (10%) pro-oxidant transcripts and 54 (29%) transcripts, which cannot be assigned clearly to either group.

The changes in transcript expression between microgravity and hypergravity samples and their respective control group were calculated (Supplementary Tables S2–S5). During parabolic flight and suborbital ballistic rocket experiments, the microgravity phase is always preceded by a hypergravity phase, including other factors such as cell cultivation in the hardware, temperature changes, and vibrations. For appropriate control, the expression changes induced by the above factors were assessed, and all of the differentially expressed transcripts were excluded from the groups of hypergravity or microgravity-sensitive transcripts. The results were shown as “controlled” versions in the respective comparisons. The transcripts were then categorized with respect to their response to different durations and qualities of gravity (controlled versions) into “no response” (no significant changes), “continuous response” (changes in the same direction compared to the initial response), “adaptation” (changes in the opposite direction compared to the initial response), and “late response” (no response after 20 s, but later significant response). We analyzed the differentially expressed genes involved in the oxidative stress-related pathways from the mentioned categories to investigate the time course of their response to different altered gravity conditions.

### 2.3. Rapid Alterations of Oxidative Stress-Related Transcripts after 20 s of Altered Gravity in Human U937 Cells

**Figure 1:**
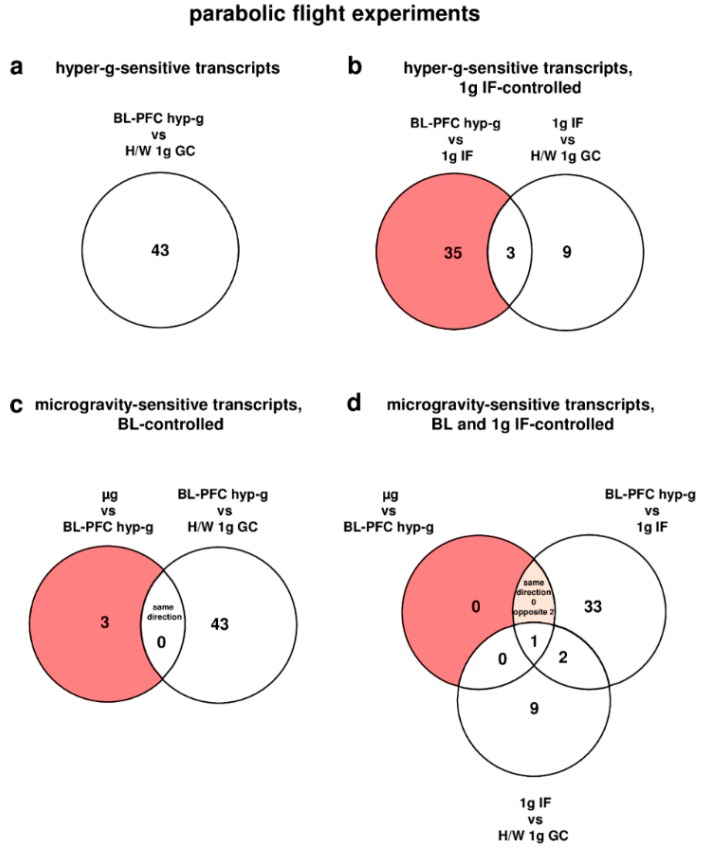
Differentially regulated transcripts (list of genes in Supplementary Table S6) of the oxidative stress response in human U937 myelomonocytic cells during the 19th DLR parabolic flight campaign. The different comparisons and resulting intersections for hypergravity and microgravity-sensitive transcripts are displayed as venn-diagramms. In the circles numbers of transcripts are given that are differentially expressed according to fold changes ≤ −1.3 or ≥ +1.3, *p* < 0.05 in the respective comparison. (**a**) Number of hyper-g sensitive transcripts. (**b**) The red area shows the number of 1g IF-controlled hyper-g-sensitive transcripts: differentially expressed in the comparison BL-PFC hyp-g versus 1g IF excluding those differentially expressed in the control comparison 1g IF versus H/W 1g GC. (**c**) The red area shows the number of BL-controlled microgravity-sensitive transcripts: differentially expressed in the comparison µg versus BL-PFC hyp-g excluding those differentially expressed in the control comparison BL-PFC hyp-g versus H/W 1g GC. (**d**) The red area shows the number of 1g IF- and BL-controlled microgravity-sensitive transcripts: differentially expressed in the comparison µg versus BL-PFC hyp-g excluding those differentially expressed in the control comparisons BL-PFC hyp-g versus 1g IF and 1g IF versus H/W 1g GC. For the abbreviations of the experiment group names see Table 1.

During the first parabola in the parabolic flight experiment, the oxidative stress-related transcripts of human U937 cells responded rapidly within 20 s of hypergravity (hyp-g) with a total number of 43 differentially regulated transcripts (comparison of experiment groups baseline/hypergravity (BL-PFC hyp-g) versus hardware (H/W) 1g ground control (GC), Table 2). After the subsequent first microgravity phase, 23 transcripts (microgravity (µg) versus H/W 1g GC) were differentially expressed, which is 1.9-fold the number of the 12 transcripts that were altered solely as a result of the flight conditions without altered gravity (comparison of 1g in-flight (IF) versus H/W 1g GC, Table 2). However, when the number of hypergravity-sensitive transcripts was determined with respect to the 1g IF control group, 38 differentially expressed transcripts were identified (Figure 1 and Table 2). Interestingly, all of the transcripts were differentially expressed in the opposite direction when comparing the different gravity conditions. After exclusion of all of the transcripts that were already altered due to flight conditions (comparison 1g IF versus H/W 1g GC), we revealed 35 hypergravity-sensitive transcripts (Figure 1). In the microgravity phase (µg versus BL-PFC hyp-g), we identified three microgravity-sensitive transcripts (Table 2 and Table 3 and Figure 1). None of the transcripts was differentially expressed in the same direction in the microgravity phase as in the hypergravity phase. The expression fold change (FC) for the hypergravity-sensitive transcripts was in the range between +3.042 (up-regulation) and −2.505 (down-regulation), with average values of +2.088 and −1.770, respectively. The expression FC for the microgravity-sensitive transcripts was in the range between +1.356 (up-regulation) and −1.495 (down-regulation) with average values of +1.347 and −1.495, respectively (Table 3).

### 2.4. Strong Response of Oxidative Stress-Related Transcripts in Human U937 Cells after 75 s of Hypergravity and 300 s of Microgravity

**Figure 2:**
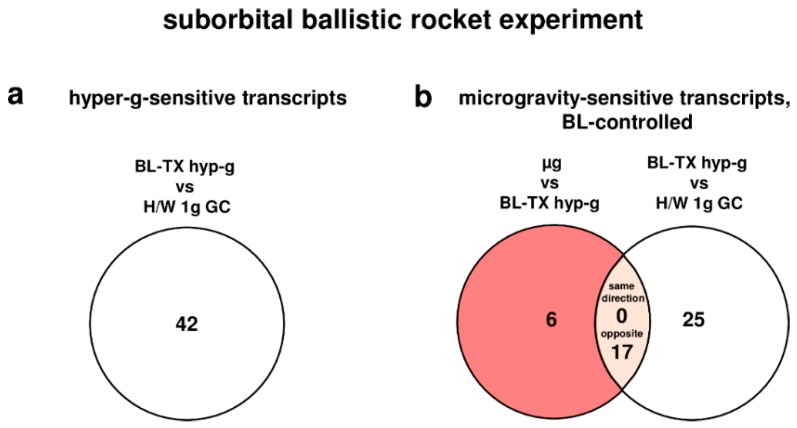
Differentially regulated transcripts (list of genes in Supplementary Table S7) of the oxidative stress response in human U937 myelomonocytic cells during the TEXUS-49 suborbital ballistic rocket campaign. The different comparisons and resulting intersections for hypergravity and microgravity-sensitive transcripts are displayed as Venn-diagramms. In the circles numbers of transcripts are given that are differentially expressed according to fold changes ≤ −1.3 or ≥ +1.3, *p* < 0.05. For the abbreviations of the experiment group names see Table 1.

During the launch phase of the suborbital ballistic rocket experiment, oxidative stress-related transcripts in human U937 cells responded rapidly within 75 s covering the hypergravity phase with a total number of 42 differentially regulated transcripts (comparison baseline/hypergravity (BL-TX hyp-g) versus H/W 1g GC, Table 4, Figure 2). After the subsequent 300 s microgravity phase, 23 transcripts were differentially expressed (µg versus BL-TX hyp-g, Table 4, Figure 2), which is 1.8-fold lower than the number of differentially expressed transcripts in hypergravity. Nine transcripts were differentially expressed in microgravity when compared to the H/W 1g ground control group (Table 4). In the investigation of hypergravity and microgravity double-sensitive transcripts (BL-TX hyp-g versus H/W 1g GC and µg versus BL-TX hyp-g), none of the transcripts in the intersection were differentially expressed in the same direction, and 17 transcripts were differentially expressed in the opposite direction (Figure 2). The expression fold change (FC) was in the range between +4.22 (up-regulation) and −1.932 (down-regulation), with average values of +1.963 and −1.656, respectively, for the hypergravity-sensitive transcripts and between +2.63 (up-regulation) and −2.237 (down-regulation) with average values of +1.727 and −1.644, respectively, for microgravity-sensitive transcripts (Table 5).

### 2.5. Oxidative Stress-Related Transcripts Were Primarily Up-Regulated in Hypergravity and Down-Regulated in Microgravity in Human U937 Cells

**Figure 3:**
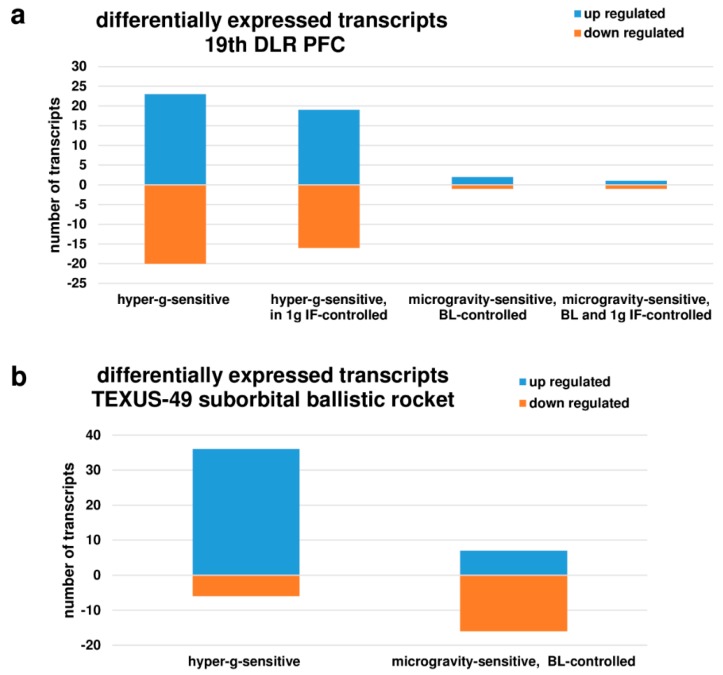
Distribution of differentially expressed transcripts of the oxidative stress response. (**a**) Hypergravity and microgravity-sensitive transcripts (10^−3^–10^−2^ g/1.8 g) identified for the 19th DLR Parabolic Flight Campaign, (**b**) Hypergravity and microgravity-sensitive transcripts (5–7 g/10^−4^ g) identified for the TEXUS-49 suborbital ballistic rocket campaign.

Figure 3 summarizes the number of differentially expressed genes in the parabolic flight and suborbital ballistic rocket experiments. Whereas up-regulation or down-regulation was almost equally distributed in differentially expressed transcripts after 20 s of altered gravity (Figure 3a), longer periods of hypergravity (75 s 5–7 g) resulted primarily in the up-regulation of transcripts and longer periods of microgravity (5 min 10^−4^ g) in the down-regulation of transcripts (Figure 3b).

### 2.6. Rapid Regulation of Oxidative Stress-Related Transcripts in Human U937 Cells

We then analyzed all of the transcripts from the parabolic flight experiments that were differentially expressed in both microgravity and hypergravity, and identified three transcripts (Table 6). All of the identified transcripts were regulated in the opposite direction in hypergravity and microgravity: transcripts that were up-regulated in hypergravity were down-regulated in microgravity, and vice versa. None of the identified transcripts was regulated in the same direction in hypergravity and microgravity (Table 6). We continued this analysis with the data from the suborbital ballistic rocket experiment, and identified 17 transcripts (Table 7) that were differentially expressed in hypergravity and microgravity, and revealed the same observation: all of the transcripts were expressed also in the opposite direction in hypergravity and microgravity, respectively (Table 7). Due to the nature of the flight profiles, the microgravity phase always followed the hypergravity phase in both flight experiments. Therefore, we investigated the hypothesis that reversely regulated transcripts are caused by counter-regulatory mechanisms. For this reason, we performed a cross-platform comparison of the hypergravity and microgravity phases, separately and with regard to their different duration. We identified nine transcripts that were altered in both platforms after 20 s and 75 s of hypergravity, respectively. We found that eight of these transcripts were regulated in the opposite direction, while only one transcript was differentially expressed in the same direction (Table 8). Thus, the same gravity condition obviously resulted in qualitatively different directions of transcript regulation as a function of time and in adapting to the new gravitational environment by counter-regulation. Cross-platform comparison of the microgravity phases revealed no common transcripts that were altered in both platforms after 20 s and 300 s of microgravity, respectively. Inter-platform comparisons of all of the transcripts that were differently regulated in hypergravity and microgravity revealed no common transcripts associated with oxidative stress response.

### 2.7. Nearly Complete Adaptation of Initially Differentially Altered Oxidative Stress-Related Transcripts in Altered Gravity in Human U937 Cells

**Figure 4:**
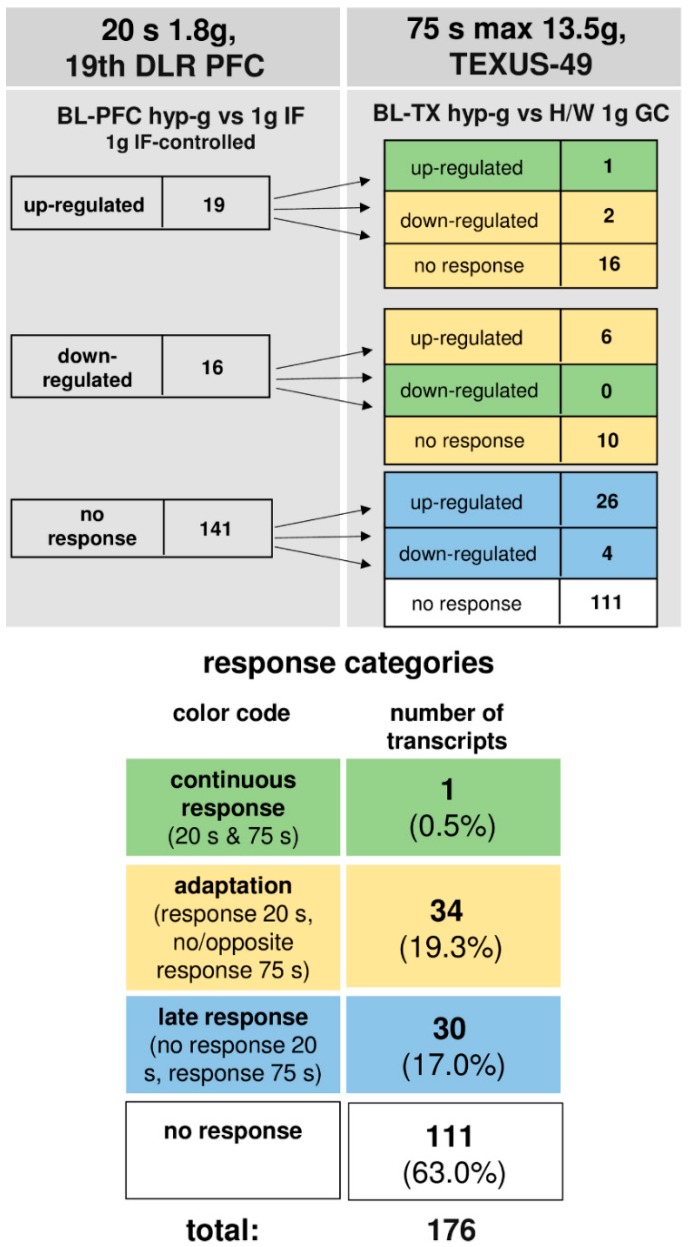
Time course of differential expression of 188 transcripts involved in oxidative stress response during hypergravity (list of genes in Supplementary Table S8). Human myelomonocytic U937 cells were exposed to 20 s and 75 s of hypergravity. The transcripts are grouped according to their regulation after the two exposure times. Continuous response means that the transcripts are either up-regulated or down-regulated at both time points. Adaptation is either the disappearance of the hypergravity-induced effect or regulation in the opposite direction after 75 s. Late response is a regulation that only appears after 75 s. Twelve transcripts out of the 188 do not appear in this scheme, as here, the maximally controlled pools are depicted where the transcripts differentially expressed in the control comparisons are eliminated. Most of the transcripts did not respond to hypergravity at either time point. For the abbreviations of the experiment group names see Table 1.

**Figure 5:**
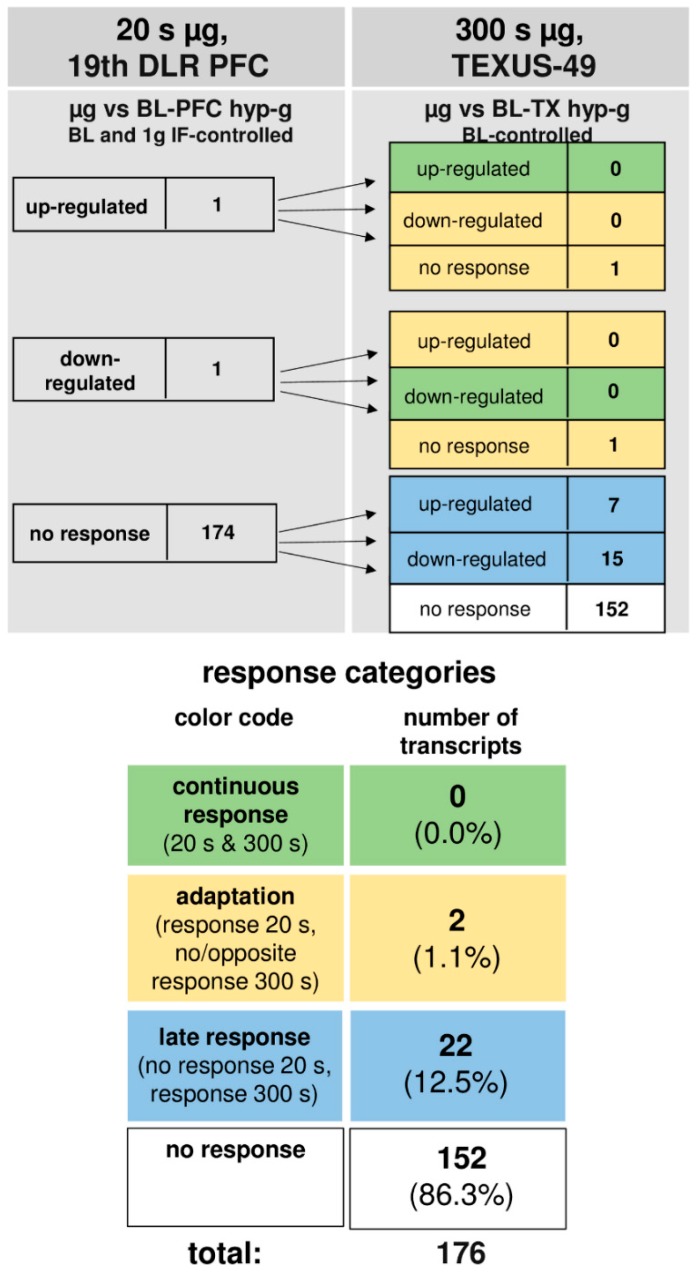
Time course of differential expression of 188 transcripts in microgravity (list of genes in Supplementary Table S9). Human myelomonocytic U937 cells were exposed to 20 s and 300 s of microgravity. The transcripts are grouped according to their regulation after the two exposure times. Continuous response means that transcripts are either up-regulated or down-regulated at both time points. Adaption is either the disappearance of the microgravity-induced effect or regulation in the opposite direction after 300 s. A late response is a regulation that only appears after 300 s. Twelve transcripts out of the 188 do not appear in this scheme, as here, the maximally controlled pools are depicted where the transcripts differentially expressed in the control comparisons are eliminated. Most of the transcripts did not respond to microgravity at either time point. For the abbreviations of the experiment group names please see Table 1.

The time course of differentially regulated transcripts in 1.8 g and 13.5 g of hypergravity is summarized in Figure 4, and that of microgravity is summarized in Figure 5. In hypergravity, a total number of 111 transcripts (63.0%) were not altered at all. Out of 35 initially altered transcripts after 20 s in 1.8 g, only one transcript (0.6%) was altered in the same direction after 75 s in a maximum of 13.5 g, whereas 34 transcripts (19.3%) adapted. In contrast, 30 transcripts (17.0%) were altered after 75 s in a maximum of 13.5 g, but not after 20 s of 1.8 g of hypergravity.

In microgravity, a total number of 152 transcripts (86.3%) were not altered at all. Out of two initially altered transcripts after 20 s, no transcript (0%) was altered in the same direction after 300 s, whereas both initially differentially expressed transcripts (1.1%) adapted. A further 22 transcripts (12.5%) were altered after 300 s, but not after 20 s of microgravity. Thus, in our experiments, which have been conducted in two different experiment platforms (19th DLR parabolic flight campaign and TEXUS-49 suborbital ballistic rocket campaign), we identified two pools of altered transcripts: a first one, which responded after seconds and adapted within 5 min at the latest, and a second one, which appeared at least after 5 min of altered gravity.

### 2.8. Nearly No Response of Oxidative Stress-Related Transcript Clusters to Altered Gravity in Human Jurkat T Cells

During the parabolic flight experiment, no oxidative stress-related transcript clusters in human Jurkat T cells responded to altered gravity during the first parabola, neither in microgravity nor in hypergravity (Table 9). During the TEXUS-51 suborbital ballistic rocket experiment, the comparisons BL-TX hyp-g versus H/W 1g GC, BL-TX hyp-g versus 1g IF and µg versus 1g IF revealed six, three, and one differentially regulated transcript clusters, respectively (Table 10). Hardware control experiments (1g IF versus H/W 1g GC) revealed 12 differently expressed transcript clusters (Table 9) in the parabolic flight hardware and 35 differently expressed transcript clusters in the TEXUS-51 hardware (Table 10), demonstrating a rapid and strong response of oxidative stress-related gene expression to changes of the cell culture environment, but not to alterations of the gravitational force. In the next step, we excluded all of the gene expression effects induced by the hardware by eliminating transcript clusters, which were significantly changed in the comparisons H/W 1g GC versus Cell Culture Control (CC) and 1g IF versus H/W 1g GC. We identified one transcript cluster as differentially expressed in hypergravity, and none in microgravity. This transcript cluster (TC11000327.hg.1; NM_001752), coding for homo sapiens catalase (CAT) mRNA was up-regulated 1.31-fold in hypergravity (comparison BL-TX hyp-g versus 1g IF).

## 3. Discussion

In a multi-platform approach, we investigated the immediate effect of altered gravity on the transcripts of 86 genes involved in ROS metabolism, antioxidative systems, and cellular response to oxidative stress (Supplementary Table S1). In human myelomonocytic U937 cells, we detected a rapid response of 19.8% of all of the investigated oxidative stress-related transcripts as early as after 20 s of hypergravity, but only of 1.1% after 20 s of microgravity. Almost all (97.1%) of the initially altered transcripts adapted after 75 s of hypergravity, and 100% after 5 min of microgravity. After almost complete adaptation of the initial pool of transcripts, a significant second pool of differentially altered transcripts appeared, consisting of 17.0% of all of the investigated transcripts after 75 s of hypergravity and of 12.5% after 5 min of microgravity. Transcripts sensitive to 75 s of hypergravity were mostly antioxidant, while the microgravity-sensitive transcripts were either anti- or pro-oxidant.

GOrilla (Gene Ontology Enrichment Analysis and Visualization tool) analysis revealed an involvement of the “multicellular organismal reproductive process” pathway by the 30 late responsive transcripts under hypergravity and of the “response to copper ions” pathway by the 22 late responsive transcripts under microgravity. Therefore, the transcripts of oxidative stress-related genes respond very quickly to altered gravity, followed by a rapid homeostatic adaptation reaction in parallel with a secondary transcript response. In contrast, we detected almost no response of oxidative stress-related transcripts in human Jurkat T cells to altered gravity. Thus, the regulation of oxidative stress-related transcripts appears to be cell-type specific.

Long-term experiments in real and “simulated” microgravity provided additional evidence of the alteration of oxidative stress-related transcripts in vivo and in vitro: a transcriptome analysis in the whole blood of astronauts after 10 days of spaceflight revealed differentially regulated transcripts in oxidative stress-related pathways [37], whereas in TK6 human lymphoblastoid cells, “simulated” microgravity induced differential expression in 370 transcripts after 48 h, which were associated with crucial biological processes such as oxidative stress response [38]. Transcript changes of oxidative stress-related genes have been reported also for HUVEC cells, which are associated with a pro-oxidative environment, inflammatory response, and a dramatical up-regulation of thioredoxin-interacting protein after 10 days of spaceflight [39], and for yeast, where 28 days of spaceflight increased oxidative stress [40]. Interestingly, the effects of 28 days of spaceflight could not be reproduced in “simulated” microgravity (rotation or random positioning machine) [40].

The rapid initial response of oxidative stress-related transcripts in U937 cells suggested a rapid transduction process between the gravitational force and a regulatory step of gene expression. Different steps of the gene expression process require different time periods. Whereas DNA decondensation occurs within minutes to hours [41], transcription factor binding requires a couple of seconds [42], and the elongation rate of RNA polymerase II ranges between 0.37 kb/min [43] and 4.3 kb/min [44], with a median rate of 2.1 kb/min [43] and a maximal theoretical velocity of above 50 kb/min [45]. Therefore, significant transcription-induced transcriptome alterations could be theoretically possible after 20 s, but only if the preceding signal cascade is fast enough and occurring within seconds or faster, as demonstrated for direct mechanotransduction into the nucleus [46,47,48]. Transcriptional effects in such a short time frame are unlikely in case of the preceding biochemical signal transduction cascade, requiring 10 min or more [49,50]. Therefore, we hypothesize that transcript alterations after 20 s of altered gravity could be the result of transcriptional regulation, provided that preceding force transduction cascades are very fast, which is unlikely for biochemical signal transduction, but very likely for direct mechanical transduction. Fast transcriptome alterations could be initiated also at the posttranscriptional level: RNA binding proteins (RBPs) bind mature mRNA and exert important regulatory effects on mRNA stability and translation. RBPs regulate mRNA degradation, stability, and half-life, and have been shown to work cooperatively with miRNAs to regulate mRNA turnover [51,52,53]. Recently, it has been demonstrated that mechanical force transduction into the chromatin can occur within seconds, subsequently resulting in the stretching and proportional accessibility of the transcriptional machinery [54] and inducing epigenetic changes in promoter regulation [55]. Therefore, it can be assumed that force-induced changes in cell geometry result in altered chromatin accessibility, based on highly precise geometric codes that are evolutionary highly conserved [56] and translate cell mechanical signals into precise genetic outputs [57].

The previously described rapid reaction and adaptation of an oxidative burst reaction [32] suggests a direct effect on the NADPH oxidase, which is a membrane-bound multiprotein complex closely associated with cytoskeletal dynamics [58,59] and mechanosensitivity [3]. Also, RhoGTPases are potential candidates to explain the structural cellular changes in microgravity [60]. In our study, we detected a significant up-regulation of p22 ^phox^ after 20 s and of p47 ^phox^ after 5 min of microgravity, whereas rac-1 was down-regulated after 5 min of microgravity. After 20 s of hypergravity, p47 ^phox^ was up-regulated and p22 ^phox^, p91 ^phox^, and p40 ^phox^ were down-regulated. After 75 s of hypergravity, p91 ^phox^ and rac-1 were up-regulated. All of the initially altered transcripts after 20 s of microgravity or hypergravity were counter-regulated afterwards, suggesting a rapid regulatory homeostatic response of the NADPH oxidase transcriptional process (Table 11).

The experiments of this study were performed in different low and high gravity environments (10^−3^–10^−2^ g/1.8 g for parabolic flight experiments and 10^−4^ g/5–7 g for TEXUS experiments) of different duration. Therefore, the duration and magnitude of the hypergravity phase could significantly affect not only the gene expression response after the hypergravity phase, but also in the subsequent microgravity phase. Currently, knowledge about the biological effects of gravitational changes in very low gravity environments is limited: whereas 2D clinostat studies with 1F6 melanoma cells suggest an effect of gravitational force changes in the low gravity environment between 0.012–0.036 g [61], no differences could be detected in NR8383 macrophages between 10^−3^–10^−2^ g during parabolic flight experiments [62] and <10^−5^ g during ISS experiments [32].

In summary, the initial transcriptional response of oxidative stress-related pathways was immediate and strong in human myelomonocytic U937 cells, but accompanied by a rapid and severe counter-regulation afterwards. In contrast, the expression of oxidative stress-related genes in human Jurkat T cells was largely insensitive to gravitational changes. Therefore, we assume a very well-regulated homeostasis and transcriptional stability of oxidative stress-related pathways in altered gravity. Since ROS are not merely a harmful by-product of respiration, but rather a highly complex and crucial signal system affecting a huge variety of cellular processes [2,63], this extensive homeostatic reaction to altered environmental gravity conditions seems very appropriate from an evolutionary perspective.

## 4. Materials and Methods

Material and Methods have been described in our previous studies [35,36].

### 4.1. Cell Culture

The human myelomonocytic cell line U937 (ATCC CRL1593.2, Manassas, VA, USA) was used as a model cell line to analyze the differential gene expression under altered gravity conditions in the human monocyte–macrophage system. U937 cells were cultured in RPMI-1640 (Biochrom/Merck Millipore, Berlin, Germany), supplemented with 10% fetal bovine serum (FBS Superior; Biochrom/Merck Millipore, Berlin, Germany), 1% glutamine (200 mM; Gibco/Life Technologies, Hessen, Germany), and 1% penicillin/streptomycin (10,000 U/mL and 10,000 µg/mL respectively; Gibco/Life Technologies, Hessen, Germany). Cells were cultured with a density of 0.2 × 10^6^ cells/mL, and medium exchange was performed every 48 h. Cells were centrifuged at 300 g for 5 min at room temperature, the supernatant was discarded, and the cell pellet was resuspended in fresh medium. An aliquot was taken and diluted with trypan blue solution, and the number of vital cells was counted. Cells were reseeded at a concentration of 0.2 × 10^6^ cells/mL in fresh medium.

Jurkat cells (ATCC Clone E6-1, TIB152™, Manassas, VA, USA) were used as a model cell line to analyze the differential gene expression under altered gravity conditions in human T cells. Jurkat T cells were cultivated in RPMI 1640 medium (Biochrom/Merck Millipore, Berlin, Germany), supplemented with 10% fetal bovine serum (FBS Superior; Biochrom/Merck Millipore, Berlin, Germany), 2 mM of glutamine (low endotoxin; Biochrom, Berlin, Germany), and 100 U/mL of penicillin, as well as 100 µg/mL of streptomycin (Biochrom, Berlin, Germany). Cells were cultured with a density of 0.2 × 10^6^ cells/mL, and medium exchange was performed every 48 h. Cells were centrifuged at 300 g for 5 min at room temperature, the supernatant was discarded, and the cell pellet was resuspended in fresh medium. An aliquot was taken, diluted with trypan blue solution, and the vital cell number was counted. Cells were reseeded at a concentration of 0.2 × 10^6^ cells/mL in fresh medium.

### 4.2. Parabolic Flight Experiment Platform

As described previously [64,65,66], parabolic flights are an ideal platform to study the initial and primary effects in mammalian cells, and the associated rapid responsive molecular alterations excluding the influences and interferences of secondary signal cascades. Parabolic flights offer a sequence of consecutive gravity conditions including 1g, 1.8 g, and microgravity (µg), with a quality of 10^−2^ g to 10^−3^ g. We designed and constructed an experimental system that allows cell culture experiments during parabolic flights on board the Airbus A300 ZERO-G (reg. no. F-BUAD) and the Airbus A310 ZERO-G (reg. no. F-WNOV), which has already been used for different parabolic flight experiments [35,36,64,65]. Primary importance was placed on realizing the direct safety technique during the development activity. The experimental structure (Supplementary Figure S1) consists of three experiment racks (storage rack for cell culture containers before the experiments at 36.5 °C, cooling rack for storage of cell culture containers after cell lysis at 4 °C, and a working rack for the handling and execution of the experiments). The modular system is able to accommodate up to 54 cell culture containers (double containment) for each flight, and allows the storage of cell cultures until the start of the experiment, the injection of a fluid (culture medium) at any defined time during the parabolic maneuver, and an automatic injection of a second fluid (lysis buffer) after 20 s at the end of a defined gravity phase. Appropriate in-flight controls were obtained during the 1-g flight phase directly before the first parabola. Injection of all of the fluids operates automatically and is pre-programmed, while the exchange of cell culture containers and supervision of the experiment was performed manually. During the 19th DLR parabolic flight campaign (PFC), we investigated the gene expression in human U937 cells in microgravity and hypergravity (1.8 g) compared to 1-g in-flight. Experiments were only conducted during the first parabola to assure that the detected differential gene expression levels were a result of the effect of gravitational change rather than an accumulated long-term effect.

### 4.3. Preparation and Execution of the Parabolic Flight Experiments

1 × 10^7^ U937 cells (during the 19th DLR PFC) or 1 × 10^7^ Jurkat T cells (during the 23rd DLR PFC) in 10 mL of medium (RPMI 1640 supplemented with 1% penicillin/streptomycin, amphotericin (Gibco/Life Technologies, Hessen, Germany), 1% glutamine, and 2% FBS (serum starved for U937 cells) or 10% FBS (Jurkat T cells) were filled into 200-mL Nutrimix bags (B. Braun Melsungen, Melsungen, Germany) and transported from the home laboratory to the pre-flight preparation laboratories at the NOVESPACE premises in Bordeaux, France. After arrival, U937 cells were de-starved with 0.8 mL of FBS per sample. Cells were stored at 36.5 °C overnight and used for the flight experiment the following morning. A temperature of 36.5 °C were chosen instead of 37 °C to rule out any thermic activation of the cells caused by the regulatory oscillation of the storage rack. For the flight day, the Nutrimix bags were placed in a solid plastic housing to create a double containment that prevented the spillage of fluids in the aircraft in case of any leakage of the hardware system.

The rapid lysis of U937 cells or Jurkat T cells in the respective gravity phase was achieved by the fast injection of five volumes of RLT buffer (Qiagen, Hilden, Germany) and mixing by inverting the samples three times immediately at the appropriate time point (1g IF samples 5 min before the first parabola, 1.8 g and microgravity samples during the first parabola). Trizol lysis (as during the suborbital ballistic rocket experiments) was not allowed during parabolic flights for safety reasons. After landing, 1-g ground controls were performed immediately using the same hardware inside the aircraft. The Supplementary Figure S1 shows a schematic overview of the individual lysis time points for the samples of the different gravity phases. Post-flight, all of the samples were directly transported to the on-site laboratory where total RNA was purified. In total, 28 U937 cell samples (6 × H/W 1g GC, 8 × 1g IF, 6 × BL-PFC hyp-g, 8 × µg, Table 1) and 24 Jurkat T cell samples (6 × 1g ground controls, 6 × 1g in-flight controls, 6 × 1.8 g and 6 × µg) were obtained.

### 4.4. RNA Isolation after the Parabolic Flight

RNA was isolated as described previously [35]. After landing of the aircraft and transport of the samples to the laboratory facilities, the protective plastic housings were disassembled, the Nutrimix bags were gently agitated, and the lysed cell solution was filled into a T75 straight neck cell culture flask. The cell solution was mixed for 10 s by vortexing and sheared by passing four times through a Ø 0.8 × 120 mm needle (B. Braun Melsungen, Melsungen, Germany) fitted to a sterile 50-mL syringe. Then, 50 mL of absolute ethanol was added, and precipitates were resuspended by vigorous shaking. A Qiavac 24 plus vacuum system (Qiagen, Hilden, Germany) was prepared by placing 24 valves and sterile connective pieces on the Qiavac 24 plus vacuum manifold, and an RNA maxi column (Qiagen, Hilden, Germany) was attached to each connective piece. The system was set to a vacuum level of −200 mbar, and the RNA maxi columns were loaded with the lysed cell suspensions. Subsequently, the valves were closed, and the RNA maxi columns were centrifuged at 3220 g for three min at room temperature. Then, 15 mL of buffer RW1 (Qiagen, Hilden, Germany) was carefully applied to the column to wash the membrane-bound RNA. After centrifugation at 3220 g for seven min at room temperature, the flow through was discarded, and an additional two washing steps were performed with 10 mL of RPE buffer (Qiagen, Hilden, Germany) followed by centrifugation at 3220 g for three min and 10 min at room temperature, respectively. The column-bound RNA was eluted by the application of 600 µL of pre-warmed RNase-free water (Qiagen, Hilden, Germany), incubation for one min at room temperature, and centrifugation for four min at 3220 g again at room temperature. The elution step was repeated with the first eluate, the column was centrifuged for seven min at 3220 g, and the purified RNA was stored in a sterile 1-mL cryotube on dry ice. Finally, the extracted RNA was transported on dry ice and stored at −80 °C until the processing of the RNA for the microarray analysis.

### 4.5. TEXUS-49 Suborbital Ballistic Rocket Experiment

TEXUS suborbital ballistic rockets consist of a two-stage VSB-30 rocket (S-30 solid rocket-stage engine with S-31 s-stage engine) and the payload. TEXUS-49 was launched on 29 March 2011 at 06:01 from the ESRANGE (European Space and Sounding Rocket Range) Space Center near Kiruna, Sweden, north of the Arctic Circle. During the ballistic suborbital flight, an altitude of 268 km and 378 s of microgravity with a quality of better than 10^−5^ g were achieved. Further parameters include: first-stage peak thrust acceleration of 6.3 g, mean thrust acceleration of 5.03 g, first-stage burnout at 12.3 s, engine separation at 13.6 s, second-stage peak thrust acceleration of 13.5 g, mean thrust acceleration of 7.30 g, burnout at 43.0 s, yo-yo despin at 56.0 s, and engine separation at 59.0 s.

### 4.6. TEXUS-51 Suborbital Ballistic Rocket Experiment

TEXUS suborbital ballistic rockets consist of a two-stage VSB-30 rocket (S-30 solid rocket-stage engine with S-31 s-stage engine) and the payload. TEXUS-51 was launched on 23 April 2015 at 09:35 from the ESRANGE (European Space and Sounding Rocket Range) Space Center near Kiruna, Sweden, north of the Arctic Circle. During the ballistic suborbital flight, an altitude of 258 km and 369 s of microgravity with a quality of better than 10^−4^ g were achieved. Further parameters include: first-stage peak thrust acceleration of 8.1 g at 2.4 s, mean thrust acceleration of 5.1 g, first-stage burnout at 12.1 s, engine separation at 13.4 s, second-stage peak thrust acceleration of 12.6 g at 34.9 s, mean thrust acceleration of 6.7 g, burnout at 43.2 s, spin at burnout of 2.8 Hz, yo-yo despin at 56.0 s, and engine separation at 59.0 s.

### 4.7. TEXUS Experiment Procedures

At ESRANGE, fully equipped laboratories enabled complete on-site preparation of the biological experiments, integration of the experiment into the payload platform 1 h before launch, and autonomous experiment execution in a programmed sequence. At the end of the free-fall period, the payload reentered the atmosphere and returned to the ground after parachute deployment at 5-km altitude and with a sink velocity of 8 m/s. The experimental unit was immediately recovered and returned to the launch site within 1.5 h after lift-off by helicopter. The general experimental composition consists of multiple sets of three syringes, filled with cell suspension (human U937 cells or human Jurkat T cells), cell culture medium, and lysis solution (Trizol LS, Life Technologies, Germany). All three syringes were connected by a T-piece, while small plugs at the outlet ports prevented any premature contact of the fluids. The syringe systems were housed in temperature-controlled, vacuum-resistant containers (Supplementary Figure S1d). The temperature-controlled syringe systems were placed at microgravity positions inside the payload structure. Before launch and during flight, syringes were activated by a pneumatic system at pre-set time points. Several pre-flight tests and development tests were conducted: biocompatibility tests, chemical stability tests, culture medium optimization with regard to buffer systems and supplements, sterilization tests, viability tests, and cell lysis tests (different lysis compounds and concentrations). The entire mission procedure was standardized and tested several times. Margins and possible holding times were determined. The experimental setup consisted of the baseline group (lysis after hypergravity phase and before onset of microgravity), in-flight microgravity group (lysis after five min of microgravity and before reentry into the Earth’s atmosphere), and 1g of ground control reference inside the experimental hardware. Cells, medium, and lysis fluid (Trizol LS) syringes were prepared directly before the launch. All of the procedures started 7 h before launch. The experimental containers were integrated into the payload structure by a “late access” port between 1:15 h and 0:45 h before launch. The sample temperature was maintained at 36.5 °C ± 0.5 °C until lysis. On landing and payload recovery, the experimental containers were immediately removed and returned to the ESRANGE laboratory for further processing. The cell suspension was transferred from the syringes into sterile plastic reaction tubes, and cells were homogenized with the subsequent isolation of RNA. The purified RNA was stored and transported on dry ice or in liquid nitrogen and analyzed afterwards by means of genome-wide expression arrays.

### 4.8. Experimental Preparation and Integration for TEXUS Experiments

U937 or Jurkat T cells were cultured in the ESRANGE laboratories on site. Cells were cultivated with a density of 0.2 × 10^6^ cells/mL, and the medium was exchanged every 48 h (see above). During the countdown phase, cells were visually inspected, harvested, the vital cell number was counted, and cells were pooled to a concentration of 5 × 10^7^ cells/mL. Then, 0.5 mL of cells (i.e., 25 million cells) were filled in sterile 3-mL plastic syringes shortly before the handover to the launch team. Additionally, a second set of syringes was filled with 0.3 mL of cell culture medium, and a third set was filled with 1-mL Trizol LS (Life Technologies, Hessen, Germany) per sample unit. The three syringes with small plugs at the outlet ports were mounted on a sterilized plastic T-block with a connecting tubing system. Experiment units were prepared and stored at 36.5 °C ± 0.5 °C until integration into the payload of the rocket or until manual execution of the ground controls, respectively. These experimental units were finally integrated into the automatically operated experiment system. During the experimental run, firstly, 0.3 mL of cell culture medium and secondly, 1 mL of Trizol LS were injected into the cell suspension at defined time points to lyse the cells and preserve the current status of differential gene expression. Directly before the µg phase, a set of samples was lysed at the time point of 75 s after launch (baseline, BL), representing the effect of hypergravity, spin, and vibrations during the launch and rocket engine burn. A second set of samples was fixed at 375 s after launch, shortly before the end of the µg phase. Additionally, 1g of ground controls were kept on the ground in the incubator analogously to the µg sample group. In total, 18 samples were obtained after the TEXUS-49 flight: 6 × H/W 1g GC, 5 × BL-TX hyp-g, 7 × µg (see Table 1). During the TEXUS-51 campaign, additionally, one sample group was installed on an integrated 1-g centrifuge, and 1-g cell culture controls were kept on the ground. In total, 39 samples were obtained after the TEXUS-51 rocket flight: 7 × 1-g ground cell culture controls (CC), 7 × 1-g hardware controls (H/W 1g GC), 9 × 1-g in-flight controls (1g IF), and 7 × BL (BL-TX hyp-g and 9 × µg (µg).

### 4.9. RNA Isolation after TEXUS Landing

The sample processing has been described previously [35]. Directly after landing, localization, and recovery of the payload by helicopter, the experiment modules were dismantled and handed over for processing. The samples containing syringes were connected to a sterile 20-G needle (B. Braun Melsungen, Melsungen, Germany), the 1.8 mL of cell suspension were sheared three times and distributed equally in two 2.0-mL reaction tubes. Then, 0.1 mL of chloroform (Sigma-Aldrich, Steinheim, Germany) was added, the homogenate was vortexed for 15 s, and then, it was incubated for five min at room temperature before a 15-min centrifugation step at 11,000 g and 4 °C. The upper phase from both 2.0-mL tubes was transferred into one 15-mL tube and 4 mL of RLT buffer (Qiagen, Hilden, Germany). Then, 3 mL of absolute ethanol was added, and the suspension was mixed. Afterwards, 4 mL of this solution was pipetted on an RNA Midi column (Qiagen, Hilden, Germany) and centrifuged for 30 s at 3000 g and room temperature. The flow through was discarded, and the residual 4 mL of RNA solution was loaded on the column. All of the samples were centrifuged for 5 min at 3000 g at room temperature. Then, the columns were washed twice with 2.5 mL of RPE buffer (Qiagen, Hilden, Germany) and centrifuged firstly for two min and additionally for five min at 3000 g at room temperature. The RNA was eluted by the addition of pre-warmed 250 µL of RNase-free water (Qiagen, Hilden, Germany) to the column; then, it was incubated for one min at room temperature, and centrifuged for three min at 3000 g and room temperature. The flow through was loaded again onto the column, incubated for one min at room temperature, and centrifuged for five min at 3000 g and room temperature. The isolated RNA was transferred into sterile 1-mL cryotubes and stored and transported at −80 °C. After arrival in the home laboratory, samples were stored at −80 °C until the processing of the RNA for the microarray analysis.

### 4.10. RNA Sample Processing and Microarray Data Analysis for U937 Cells

The RNA quantity and quality of the samples of the 19th DLR parabolic flight campaign and the TEXUS-49 sounding rocket mission were analyzed using a Nanodrop 1000 (Thermo Scientific). All of the RNA samples were of high quality with 260/280 nm ratios between 1.9 and 2.1. The RNA integrity number (RIN) was measured using an Agilent 2100 Bioanalyzer (Agilent Technologies, Santa Clara, CA, USA), and only RNA samples with a RIN of >8.7 were used for the following microarray analysis. Then, 400 ng of total RNA were cy3-labeled with the “Low RNA Input Linear Amplification Kit, PLUS, One-Color“ (Agilent Technologies) and hybridized for 17.5 h to a NimbleGen expression microarray (12 × 135,000 features) applying the “Gene Expression Hybridization Kit” (Agilent Technologies, USA). Microarrays were washed and scanned with the Micro Array Scanner G2505B (Agilent Technologies, USA). The image files of the scanner were analyzed with the NimbleScan Software 2.6 using the Robust Multi-Array Analysis (RMA) with default parameters. RMA represents a probe-level summarization method that identifies probes that are outliers in the overall behavior of the expression measured for a given gene. The differential expression of transcripts was determined based on the normalized microarray data using Excel 2013 and expression fold changes (FCs) of all of the transcripts on the microarray were calculated. FCs were used for comparisons of experimental groups within one experimental platform. For this, averages of the linear expression values were determined for each experimental group. The ratio was calculated by dividing the average value of one experimental group by the average value of the experimental group to which it should be compared. If the ratio is ≥1, it is equal to the FC; if the ratio is <1, the FC was determined by building the negative reciprocal of the ratio. Furthermore, *t*-tests were performed for all of the comparisons. FCs ≤ −1.3 or ≥ 1.3 with a *p*-value of < 0.05 were regarded as representing a significantly differential expression.

### 4.11. RNA Sample Processing and Microarray Data Analysis for Jurkat T Cells

Gene expression profiling was performed using Affymetrix GeneChip^®^ Human Transcriptome Array 2.0 (Affymetrix United Kingdom Ltd., High Wycombe, UK) containing 44,699 protein coding genes and 22,829 non-protein coding genes. RNA quantity and quality were determined by measurement of concentration with absorbance at 260 nm and 280 nm (NanoDrop 2000c; Thermo, Fisher Scientific, Bonn, Germany) and by means of an Agilent 2100 Bioanalyzer with an RNA 6000 Nano kit and 2100 Expert software (version B.02.07) (all Agilent Technologies Deutschland GmbH, Boeblingen, Germany) at the Core Facility Genomics of the Medical Faculty Muenster (Muenster, Germany). Only high-quality RNA with 260/280 nm ratios between 1.97–2.04 and RNA integrity numbers (RIN) >8.2 was used for further microarray analysis. The fragmented and biotinylated DNA targets were prepared according to the standard Affymetrix WT PLUS Reagent Kit protocol (Affymetrix GeneChip^®^ WT PLUS Reagent Kit, 902,280) from 100 ng of total RNA starting material and 5.5 µg of cDNA intermediate product. DNA targets were hybridized for 17 h at 45 °C on GeneChip Human Transcriptome Arrays 2.0. GeneChips were washed and stained in the Affymetrix Fluidics Station 450 according to the standard GeneChip Expression Wash, Stain, and Scan protocol (Affymetrix GeneChip Wash, Stain and Scan Kit, 900,720). Subsequently, the GeneChips were scanned using the Affymetrix 3000 7G scanner. For microarray data analysis, the Affymetrix Expression Console and Transcriptome Analysis Console was used. The robust multi-array (RMA) averaging method was applied for background correction, quantile normalization, and probe summarization. After background correction, the base2 logarithm of each background-corrected perfect match intensity was obtained. These background-corrected and log-transformed perfect match intensities were normalized using the quantile normalization method developed by Bolstad et al. [67]. In the quantile normalization method, the highest background-corrected and log-transformed perfect match intensity on each GeneChip is determined. These values were averaged, and the individual values were replaced by the average. This process was repeated with what were originally the second-highest background-corrected and log-transformed perfect match intensities on each GeneChip, the third highest, etc. Gene expression differences were determined by applying an analysis of variance (ANOVA). Genes of interest were identified, and the log2 values of the measured fluorescent intensities returned by the Affymetrix Expression Console were back calculated to linear values. Then, the means of all of the values of the same gene generated by different probes were calculated.

### 4.12. Selection of Array Probes for Analysis of Oxidative Stress Response

A list of 86 genes relevant for oxidative stress response was compiled on the basis of the gene collection of the RT² Profiler™ PCR Array Human Oxidative Stress (Qiagen, Hilden, Germany) and literature [68]. All of the transcripts from the NimbleGen expression microarray and transcript clusters from the Affymetrix GeneChip^®^ Human Transcriptome Array matching in gene symbol and Entrez ID Number (current assignment) were selected. In case the gene symbol and Entrez ID within the annotation of the arrays were different from the current annotation, the probe was only included if validated by the current annotation. All of the selected transcripts and transcript clusters are shown in Supplementary Table S1.

### 4.13. Intra-Platform and Inter-Platform Comparisons

To further validate the relation between the different gravitational conditions and the detected differential expressions, intersections were made between the experimental comparison and respective control comparison whenever available. Transcripts that were differentially expressed in the experimental and the control comparison were consequently excluded from the pools of gravisensitive transcripts. To analyze whether the transcripts respond preferentially to hypergravity, microgravity, or to both conditions, intersections were made between the pools of hypergravity and microgravity-sensitive transcripts. Furthermore, to evaluate the gravisensitivity of transcripts over time, intersections were made between the pools of gravisensitive transcripts of both experimental platforms.

### 4.14. Data Availability

The datasets generated and analyzed during the current study can be accessed in the GEO (Gene Expression Omnibus) repository (www.ncbi.nlm.nih.gov/projects/geo) under accession no. GSE101309 (U937 cells datasets) and accession no. GSE94256 (Jurkat T cell datasets).

## Figures and Tables

**Table 1:** Abbreviations of experiment groups of the 19th DLR and 23rd DLR parabolic flight campaign and TEXUS-49 and TEXUS-51 suborbital ballistic rocket campaigns. Additional information on the group is given in squared brackets.

		19th DLR PFC 23rd DLR PFC	TEXUS-49 TEXUS-51
**Gravity Condition**	Cell Culture Control	n/a	CC (TEXUS-51)
	Hardware 1g Ground Control	H/W 1g GC	H/W 1g GC
	1g In-Flight	1g IF	1g IF (TEXUS-51)
	Baseline/Hypergravity [Directly Before µg Phase]	BL-PFC hyp-g (1.8 g, 20 s)	BL-TX hyp-g (max. 13.5 g, 75 s)
	Microgravity (µg)	µg (20 s)	µg (300 s)

DLR: German Aerospace Center; PFC: parabolic flight campaign.

**Table 2:** Numbers of transcripts that are differentially expressed in response to altered gravitational conditions in the 19th DLR parabolic flight campaign. Transcripts that were significantly changed in the control comparisons were eliminated. Differential expression is defined as *t*-test *p*-value < 0.05; fold change (FC) ≤ −1.3 or ≥ +1.3. FCs are ratios between the averages of linear expression values. If the ratio is <1, FC is calculated as the negative reciprocal of the ratio. For the abbreviations of the experiment group names see Table 1.

		1g IF vs. H/W 1g GC	BL-PFC hyp-g vs. 1g IF	µg vs. BL-PFC hyp-g	µg vs. 1g IF	BL-PFC hyp-g vs. H/W 1g GC	µg vs. H/W 1g GC
**Differentially Expressed Transcripts**	up-regulated	7	20	2	0	23	10
	down-regulated	5	18	1	2	20	13
	total number	12	38	3	2	43	23

**Table 3:** Number of differentially expressed transcripts in comparisons of the experiment groups of the 19th DLR parabolic flight campaign. Differential expression is defined as *t*-test *p*-value < 0.05; fold change (FC) ≤ −1.3 or ≥ +1.3. If the ratio is <1, FC is calculated as the negative reciprocal of the ratio. The term “transcripts” refers to 188 oxidative stress-related transcripts, representing 86 genes. For the abbreviations of the experiment group names see Table 1.

**Hypergravity-Sensitive Transcripts**				
		Differentially expressed in BL-PFC hyp-g vs. H/W 1g GC (TEXUS-49-analogous)	Average FC of BL-PFC hyp-g vs. H/W 1g GC	Min/Max FC of BL-PFC hyp-g vs. H/W 1g GC
**Differentially Expressed Transcripts**	up-regulated	23	2.088	3.042
	down-regulated	20	−1.770	−2.505
	total number	43		
**Hypergravity-Sensitive Transcripts, 1g IF-Controlled**				
		Differentially expressed in BL-PFC hyp-g vs. 1g IF, but not differentially expressed in 1g IF vs. H/W 1g GC	Average FC of BL-PFC hyp-g vs. 1g IF	Min/Max FC of BL-PFC hyp-g vs. 1g IF
**Differentially Expressed Transcripts**	up-regulated	19	1.727	2.184
	down-regulated	16	−1.590	−2.014
	total number	35		
**Microgravity-Sensitive Transcripts, BL-Controlled**				
		Differentially expressed in µg vs. BL-PFC hyp-g, but not differentially expressed in the same direction in BL-PFC hyp-g vs. H/W 1g GC (TEXUS-49-analogous)	Average FC of µg vs. BL-PFC hyp-g	Min/Max FC of µg vs. BL-PFC hyp-g
**Differentially expressed transcripts**	up-regulated	2	1.347	1.356
	down-regulated	1	−1.495	−1.495
	total number	3		
**Microgravity-Sensitive Transcripts, BL and 1g IF-Controlled**				
		Differentially expressed in µg vs. BL-PFC hyp-g, but not differentially expressed in 1g IF vs. H/W 1g GC or in the same direction in BL-PFC hyp-g vs. 1g IF	Average FC µg vs. BL-PFC hyp-g	Min/Max µg vs. BL-PFC hyp-g
**Differentially expressed transcripts**	up-regulated	1	1.338	1.338
	down-regulated	1	−1.495	−1.495
	total number	2		

**Table 4:** Number of differentially expressed transcripts in comparisons of the experiment groups of the TEXUS-49 suborbital ballistic rocket campaign. Differential expression is defined as *t*-test *p*-value < 0.05; fold change (FC) ≤ −1.3 or ≥ +1.3. If the ratio is <1, FC is calculated as the negative reciprocal of the ratio. The term “transcripts” refers to 188 oxidative stress-related transcripts of the array, representing 86 genes. For the abbreviations of the experiment group names see Table 1.

		µg vs. BL-TX hyp-g	BL-TX hyp-g vs. H/W 1g GC	µg vs. H/W 1g GC
**Differentially Expressed Transcripts**	up-regulated	7	36	3
	down-regulated	16	6	6
	total number	23	42	9

**Table 5:** Numbers of transcripts that are differentially expressed in response to altered gravitational conditions in the TEXUS-49 suborbital ballistic rocket campaign. Transcripts that were significantly changed in the control comparisons were eliminated. Differential expression is defined as *t*-test *p*-value < 0.05; fold change (FC) ≤ −1.3 or ≥ +1.3. FCs are ratios between the averages of linear expression values. If the ratio is <1, FC is calculated as the negative reciprocal of the ratio. For the abbreviations of the experiment group names see Table 1.

**Hypergravity-Sensitive Transcripts**				
		Differentially expressed in BL-TX hyp-g vs. H/W 1g GC	Average FC BL-TX hyp-g vs. H/W 1g GC	Min/Max FC BL-TX hyp-g vs. H/W 1g GC
**Differentially Expressed Transcripts**	up-regulated	36	1.963	4.22
	down-regulated	6	−1.656	−1.932
	total number	42		
**Microgravity-sensitive transcripts, BL-controlled**				
		Differentially expressed in µg vs. BL TX hyp-g, but not differentially expressed in the same direction in BL-TX hyp-g vs. H/W 1g GC	Average FC µg vs. BL-TX hyp-g	Min/Max FC µg vs. BL-TX hyp-g
**Differentially Expressed Transcripts**	up-regulated	7	1.727	2.630
	down-regulated	16	−1.644	−2.237
	total number	23		

**Table 6:** Differentially regulated transcripts identified for the parabolic flight data sets. All of the transcripts were reversely regulated with respect to microgravity and hypergravity conditions. Plus: significantly up-regulated transcripts (*p*-value < 0.05, fold change (FC) ≥ +1.3); minus: significantly down-regulated transcripts (*p*-value < 0.05, FC ≤ −1.3). FCs are ratios between the averages of the linear expression values. If the ratio is <1, FC is calculated as the negative reciprocal of the ratio. For the abbreviations of the experiment group names see Table 1.

		19th DLR PFC Fold Change			
		BL-PFC hyp-g vs. 1g IF		µg vs. BL-PFC hyp-g	
**Gene Name**	Probeset ID	FC	*p*-value	FC	*p*-value
**CYBA**	BC006465	−1.382	0.0093	+1.338	0.0156
**PTGS1**	DQ180741	−1.636	0.0019	+1.356	0.0292
**PXDN**	XM_056455	+1.655	0.0062	−1.495	0.0187

**Table 7:** Differentially regulated transcripts identified for the TEXUS-49 suborbital ballistic rocket campaign data sets. All 17 transcripts were reversely regulated with respect to microgravity and hypergravity conditions. Plus: significantly up-regulated transcripts (*p*-value < 0.05, fold change (FC) ≥ +1.3), minus: significantly down-regulated transcripts (*p*-value < 0.05, FC ≤ −1.3). FCs are ratios between the averages of linear expression values. If the ratio is <1, FC is calculated as the negative reciprocal of the ratio. For the abbreviations of the experiment group names see Table 1.

		TEXUS-49 Fold Change	
Gene Name	Probeset ID	BL-TX hyp-g vs. H/W 1g GC	µg vs. BL-TX hyp-g
ALOX12	NM_000697	−1.602	+1.537
GCLM	BC041809	+2.711	−2.118
GSR	BC069244	+1.632	−1.394
GSR	NM_000637	+1.831	−1.625
MSRA	BC054033	+1.474	−1.493
MSRA	AY958431	+1.517	−1.535
MSRA	NM_012331	+1.526	−1.555
MT3	BC013081	−1.789	+1.808
OXSR1	BC008726	+1.530	−1.701
PRDX4	NM_006406	+1.545	−1.356
PRDX4	BC003609	+1.567	−1.417
PRDX4	BC016770	+1.590	−1.419
PRDX4	BC007107	+1.611	−1.468
PRNP	NM_000311	+2.233	−2.237
PTGS2	NM_000963	+1.556	−1.482
SELS	NM_203472	+1.731	−1.856
SOD1	NM_000454	+2.429	−2.099

**Table 8:** Differentially regulated transcripts in hypergravity identified in the 19th DLR parabolic flight and TEXUS-49 suborbital ballistic rocket campaigns. Eight transcripts (belonging to six genes) were reversely regulated with respect to both altered gravity platforms. One transcript (belonging to the gene PTGS2) was significantly up-regulated in both campaigns. Plus: significantly up-regulated transcripts (*p*-value < 0.05, fold change (FC) ≥ 1.3), minus: significantly down-regulated transcripts (*p*-value < 0.05, FC ≤ −1.3). FCs are ratios between the averages of linear expression values. If the ratio is <1, FC is calculated as the negative reciprocal of the ratio. Remark: The FCs for BC032720 were −1.81890805409698 (BL-PFC hyp-g versus 1g IF) and 1.81896050075992 (BL-TX hyp-g versus H/W 1g GC). For the abbreviations of the experiment group names see Table 1.

Gene Name	Full Gene Name	Probeset ID	Gene Function Adopted from GeneCards HUMAN GENE DATABASE	Fold Change 19th DLR PFC BL-PFC hyp-g vs. 1g IF	Fold Change TEXUS-49 BL-TX hyp-g vs. H/W 1g GC
CYBB	Cytochrome B-245 Beta Chain, NADPH Oxidase 2, GP91-PHOX	BC032720	Primary component of the microbicidal oxidase system of phagocytes	−1.819	+1.819
		NM_000397		−1.808	+1.794
GCLM	Glutamate-Cysteine Ligase Modifier Subunit	NM_002061	First-rate limiting enzyme of glutathione synthesis	−1.810	+4.220
GPX5	Glutathione Peroxidase 5	NM_003996	Protects cells and enzymes from oxidative damage by catalyzing the reduction of hydrogen peroxide, lipid peroxides, and organic hydroperoxide by glutathione.	+1.494	−1.741
GSR	Glutathione-Disulfide Reductase	BC069244	Maintains high levels of reduced glutathione in the cytosol	−1.527	+1.632
		NM_000637		−1.542	+1.831
KRT1	Keratin 1	BC063697	May regulate the activity of kinases such as PKC and SRC via binding to integrin beta-1 (ITB1) and the receptor of activated protein C kinase 1 (RACK1)	+2.077	−1.529
PTGS2	Prostaglandin-Endoperoxide Synthase 2, Cyclooxygenase-2	AJ634912	Converts arachidonate to prostaglandin H2 (PGH2), production of inflammatory prostaglandins, associated with increased cell adhesion and resistance to apoptosis.	+1.414	+2.253
SOD1/2	Superoxide Dismutase1/2	NM_000636	Destroys superoxide anion radicals	−1.946	+2.517

PKC: *protein kinase C*.

**Table 9:** Differentially expressed oxidative stress-related transcript clusters in T cells during the 23rd parabolic flight campaign. Number of significantly differentially expressed transcript clusters that were up-regulated or down-regulated in the respective comparison. Differential expression is defined as *t*-test *p*-value < 0.05; fold change (FC) < −1.3 or > +1.3. FCs are ratios between the averages of linear expression values. If the ratio is <1, FC is calculated as the negative reciprocal of the ratio. A total of 106 transcript clusters, belonging to 86 genes involved in oxidative stress response, were investigated. For the abbreviations of the experiment group names see Table 1.

	1g IF vs. H/W 1g GC	BL-PFC hyp-g vs. 1g IF	μg vs. 1g IF	μg vs. BL-PFC hyp-g
**Up-Regulated Transcript Clusters**	10	0	0	0
**Down-Regulated Transcript Clusters**	2	0	0	0
**Total Number of Differentially Expressed Transcript Clusters**	12	0	0	0

**Table 10:** Differentially expressed oxidative stress-related transcript clusters in Jurkat T cells during the TEXUS-51 mission. The number of significantly differentially expressed transcript clusters that were up-regulated or down-regulated in the respective comparison. Differential expression is defined as a *t*-test *p*-value < 0.05; fold change (FC) < −1.3 or > +1.3. FCs are ratios between the averages of linear expression values. If the ratio is <1, FC is calculated as the negative reciprocal of the ratio. A total of 106 transcript clusters, belonging to 86 genes involved in oxidative stress response, were investigated. For the abbreviations of the experiment group names see Table 1.

	H/W 1g GC vs. CC	BL-TX hyp-g vs. H/W 1g GC	BL-TX hyp-g vs. 1g IF	µg vs. 1g IF	µg vs. BL-TX hyp-g
**Up-Regulated Transcript Clusters**	22	6	3	1	0
**Down-Regulated Transcript Clusters**	13	0	0	0	0
**Total Number of Differentially Expressed Transcript Clusters**	35	6	3	1	0

**Table 11:** Transcripts belonging to genes coding for nicotinamide adenine dinucleotide phosphate (NADPH) oxidase subunits. Plus values with asterix (*): significantly up-regulated transcripts (*p*-value < 0.05, fold change (FC) ≥ 1.3), minus values with asterix (*): significantly down-regulated transcripts (*p*-value < 0.05, FC ≤ −1.3). For the abbreviations of the experiment group names see Table 1.

			19th DLR PFC		TEXUS-49	
Probeset ID	Gene Name	Alternative Gene Name	Fold Change BL-PFC hyp-g vs. 1g IF	Fold Change µg vs. BL-PFC hyp-g	Fold Change BL-TX hyp-g vs. H/W 1g GC	Fold Change µg vs. BL-TX hyp-g
BC006465	CYBA	p22 ^phox^	−1.381 *	+1.338 *	−1.052	+1.026
NM_000101	CYBA	p22 ^phox^	−1.411 *	+1.283	−1.055	+1.061
BC032720	CYBB	p91 ^phox^	−1.818 *	+1.365	+1.818 *	−1.971
NM_000397	CYBB	p91 ^phox^	−1.807 *	+1.361	+1.793 *	−1.956
NM_000265	NCF1	p47 ^phox^	+1.427 *	−1.264	−1.248	+1.328 *
XM_928908	NCF1	p47 ^phox^	+1.498 *	−1.272	−1.356	+1.726 *
BC001606	NCF2	p67 ^phox^	−1.265	+1.179	−1.052	−1.529
NM_000433	NCF2	p67 ^phox^	−1.231	+1.171	−1.189	−1.305
NM_000631	NCF4	p40 ^phox^	−1.432 *	+1.345	−1.238	−1.248
BC004247	RAC1		+1.076	−1.030	+1.130	−1.005
NM_006908	RAC1		+1.094	−1.062	+1.993 *	−1.524 *
NM_002872	RAC2		−1.083	+1.007	+1.013	−1.024

## Supplementary Materials

Supplementary materials can be found at http://www.mdpi.com/1422-0067/19/9/2814/s1.

## Abbreviations

DLR	German Aerospace Center
ESRANGE	European Space and Sounding Rocket Range
FC	Fold change
RIN	RNA Integrity Number
TEXUS	Technologische Experimente unter Schwerelosigkeit
