# Supplementary material for: Transcriptional Homeostasis of Oxidative Stress-Related Pathways in Altered Gravity
Source: Int J Mol Sci. 2018 Sep 18;19(9):2814. doi: 10.3390/ijms19092814 (PMC6164947; doi:10.3390/ijms19092814)
Supplement: Supplementary file 1 [file ijms-19-02814-s001.pdf]

Supplementary Figure 1. Experiment design of the parabolic flight (19th and 23rd DLR PFC) and suborbital ballistic rocket (TEXUS-49 and TEXUS-51) experiments. **(a)** During the parabolic flight campaigns, U937 or Jurkat T cells were analyzed before and during the first parabola. 1g in-flight control (1g IF) samples were lysed 5 min before the first parabola, 1.8g hypergravity samples at the end of the first 1.8g phase, and microgravity samples at the end of the first microgravity phase. After the flight, 1g ground controls were performed in the experiment hardware inside the aircraft. **(b)** During the suborbital ballistic rocket missions, U937 and Jurkat T cells were lysed at time point T+75s to monitor the hypergravity and vibration effects of the rocket launch. Microgravity samples and 1g in-flight reference centrifuge control samples (for the TEXUS-51 mission) were lysed after 375s post-launch. Additionally, hardware 1g ground controls as well as cell culture controls (for the TEXUS-51 mission) were lysed post-flight approximately 15min after the rocket launch. **(c)** In-flight experiment system for parabolic flights on board the Airbus A300 / A310 ZERO-G. **(c1)** Experiment hardware structure which consists of an incubator rack to store the cell containers at 37°C before the experiment (1), an experiment rack, in which all technical aggregates are accommodated for the execution of the experiment and where the living cells are processed during altered gravity (2), and a cooling rack to store all cell containers at 4°C after the injection of the lysis solution until landing (3). **(c2)** Double-walled, liquid-proof cell container. A maximum of 54 container can be accommodated during one flight. 1 = plastic container, 2 = air valve, 3 = internal sterile cell culture bag (Nutrimix, 0.25 l), 4 = connector 1 (medium), 5 = connector 2 (lysis buffer), 6 = connector 3 (port for filling of cells, performed pre-flight), 7 = plastic flange. **(d)** In-flight experiment system for the suborbital ballistic rocket flight of the TEXUS-49 and TEXUS-51 payload. TEXUS consists of a VSB-30 engine (not shown) and of the payload structure. Sets of three sterile syringes were filled with cell suspension, medium, and lysis buffer connected by a T-piece with small plugs at the outlet ports to prevent premature contact of the fluids **(d4)**. The syringe systems are accommodated in tempered and vacuum-resistant containers **(d3,** d5) at the static (d2) or centrifuge (d1) (TEXUS-51) position. The figure has been re-used and modified from our previous work [35,36] under the CC BY license (Creative Commons Attribution 4.0 International License).

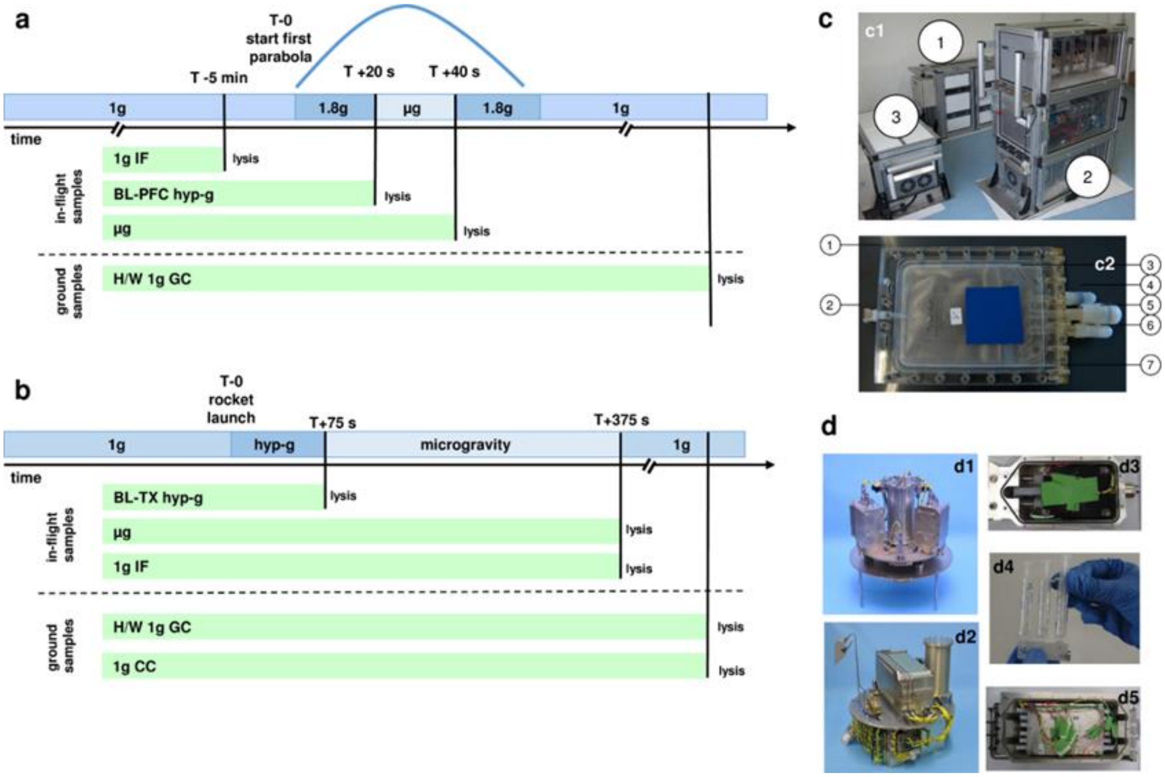

Supplementary Table 1. Selection of microarray probes for the oxidative stress response analysis. Based on the gene collection of the RT² Profiler™ PCR Array Human Oxidative Stress (Qiagen) and further literature [67] 86 genes relevant for oxidative stress response were identified and collected. For each gene, the gene symbol, as well as the UniGene, GenBank and Entrez ID identifier are listed. Regarding the Affymetrix array, the number of transcript clusters and the associated transcript cluster IDs are mentioned for each gene. For the NimbleGen array, the probeset IDs as well as the number of transcripts are given.

				**Affymetrix GeneChip® Human Transcriptome Array**		**NimbleGen Array**	
**Symbol**	**UniGene**	**GenBank**	**Entrez ID**	**Number of transcript clusters**	**Transcript Cluster IDs**	**Number of transcripts**	**Probeset IDs**
ALB	Hs.418167	NM_000477	213	1	TC04000403.hg.1	4	BC034023; BC035969; BC041789; NM_000477
ALOX12	Hs.654431	NM_000697	239	1	TC17000081.hg.1	2	BC069557; NM_000697
AOX1	Hs.406238	NM_001159	316	1	TC02001162.hg.1	1	NM_001159
APOE	Hs.654439	NM_000041	348	1	TC19000633.hg.1	1	NM_000041
ATOX1	Hs.125213	NM_004045	475	1	TC05001957.hg.1	2	BC112248; NM_004045
BNIP3	Hs.144873	NM_004052	664	1	TC10001769.hg.1	2	BC021989; NM_004052
CAT	Hs.502302	NM_001752	847	1	TC11000327.hg.1	2	BC112217; NM_001752
CCL5	Hs.514821	NM_002985	6352	1	TC17001378.hg.1	2	BC008600; NM_002985
CCS	Hs.502917	NM_005125	9973	1	TC11000672.hg.1	2	BC105016; NM_005125
CYBA	Hs.513803	NM_000101	1535	1	TC16001339.hg.1	2	BC006465; NM_000101
CYBB	Hs.292356	NM_000397	1536	1	TC0X000171.hg.1	2	BC032720; NM_000397
CYGB	Hs.95120	NM_134268	114757	1	TC17001902.hg.1	1	NM_134268
DHCR24	Hs.498727	NM_014762	1718	1	TC01002694.hg.1	2	BC004375; NM_014762
DUOX1	Hs.272813	NM_175940	53905	1	TC15000352.hg.1	1	NM_017434
DUOX2	Hs.71377	NM_014080	50506	1	TC15001305.hg.1	2	AF230496; NM_014080
DUSP1	Hs.171695	NM_004417	1843	1	TC05002066.hg.1	1	NM_004417

EPHX2	Hs.212088	NM_001979	2053	1	TC08000208.hg.1	1	NM_001979
EPX	Hs.279259	NM_000502	8288	1	TC17000711.hg.1	1	NM_000502
FOXM1	Hs.239	NM_021953	2305	1	TC12001106.hg.1	3	BC006192; NM_021953; NM_202003
FTH1	Hs.645560	NM_002032	2495	1	TC11001860.hg.1	3	BC000857; BC066341; NM_002032
GCLC	Hs.654465	NM_001498	2729	1	TC06004051.hg.1	2	BC039894; NM_001498
GCLM	Hs.315562	NM_002061	2730	1	TC01002882.hg.1	2	BC041809; NM_002061
GPX1	Hs.76686	NM_000581	2876	1	TC03001407.hg.1	3	BC000742; NM_000581; NM_201397
GPX2	Hs.2704	NM_002083	2877	1	TC14001223.hg.1	2	BC005277; NM_002083
GPX3	Hs.386793	NM_002084	2878	1	TC05000837.hg.1	2	BC050378; NM_002084
GPX4	Hs.433951	NM_002085	2879	1	TC19000030.hg.1	2	NM_001039847; NM_001039848
GPX5	Hs.248129	NM_001509	2880	2	TC06000286.hg.1; TC6_cox_hap2000001.hg.1	2	NM_001509; NM_003996
GPX6	Hs.448570	NM_182701	257202	2	TC06001444.hg.1; TC6_cox_hap2000110.hg.1	1	NM_182701
GPX7	Hs.43728	NM_015696	2882	1	TC01000643.hg.1	2	BC032788; NM_015696
GSR	Hs.271510	NM_000637	2936	1	TC08001110.hg.1	2	BC069244; NM_000637
GSS	Hs.82327	NM_000178	2937	1	TC20000789.hg.1	1	NM_000178
GSTP1	Hs.523836	NM_000852	2950	1	TC11000694.hg.1	2	BC010915; NM_000852
GSTZ1	Hs.655292	NM_001513	2954	1	TC14000489.hg.1	3	BC001453; NM_001513; NM_145870
GTF2I	Hs.520459	NM_001518	2969	1	TC07000458.hg.1	2	BC004472; NM_001518
HMOX1	Hs.517581	NM_002133	3162	1	TC22000259.hg.1	2	BC001491; NM_002133

HSPA1A	Hs.728810	NM_005345	3303	15	TC06001547.hg.1; TC6_apd_hap1000088.hg.1; TC6_cox_hap2000174.hg.1; TC6_dbb_hap3000163.hg.1; TC6_qbl_hap6000164.hg.1; TC06000384.hg.1; TC6_apd_hap1000039.hg.1; TC6_cox_hap2000078.hg.1; TC6_dbb_hap3000069.hg.1; TC6_qbl_hap6000069.hg.1; TC06000385.hg.1; TC6_apd_hap1000040.hg.1; TC6_cox_hap2000079.hg.1; TC6_dbb_hap3000070.hg.1; TC6_qbl_hap6000070.hg.1	3	AK096017; BC009322; NM_005345
KRT1	Hs.80828	NM_006121	3848	1	TC12001532.hg.1	2	BC063697; NM_006121
LPO	Hs.234742	NM_006151	4025	1	TC17000712.hg.1	2	BC107166; NM_006151
MB	Hs.517586	NM_005368	4151	2	TC22000697.hg.1; TC22000698.hg.1	4	BC018001; NM_005368; NM_203377; NM_203378
MBL2	Hs.499674	NM_000242	4153	1	TC10001301.hg.1	2	BC069338; NM_000242
MGST3	Hs.191734	NM_004528	4259	2	TC01001444.hg.1; TC01001443.hg.1	2	CR607567; NM_004528
MPO	Hs.458272	NM_000250	4353	1	TC17001727.hg.1	2	NM_000250; X04876
MPV17	Hs.75659	NM_002437	4358	1	TC02001681.hg.1	1	NM_002437
MSRA	Hs.490981	NM_012331	4482	2	TC08000071.hg.1; TC08000073.hg.1	3	AY958431; BC054033; NM_012331
MT3	Hs.73133	NM_005954	4504	1	TC16000465.hg.1	2	BC013081; NM_005954
NCF1	Hs.647047	NM_000265	653361	1	TC07000459.hg.1	2	NM_000265; XM_928908
NCF2	Hs.587558	NM_000433	4688	1	TC01003616.hg.1	2	BC001606; NM_000433
NCF4	Hs.474781	NM_000631	4689	1	TC22000270.hg.1	1	NM_000631
NOS2	Hs.709191	NM_000625	4843	1	TC17002894.hg.1	1	NM_000625
NOX4	Hs.371036	NM_016931	50507	1	TC11002178.hg.1	4	AJ704727; BC040105; BC051371; NM_016931
NOX5	Hs.657932	NM_024505	79400	1	TC15000630.hg.1	2	AK074071; NM_024505
NQO1	Hs.406515	NM_000903	1728	1	TC16001225.hg.1	2	BC007659; NM_000903

NUDT1	Hs.534331	NM_002452	4521	1	TC07000029.hg.1	6	BC014618; BC065367; NM_002452; NM_198948; NM_198949; NM_198953
OXR1	Hs.148778	NM_181354	55074	1	TC08000660.hg.1	5	AB075503; AF309387; AK096148; BC032710; NM_181354
OXSR1	Hs.475970	NM_005109	9943	1	TC03000188.hg.1	2	BC008726; NM_005109
PDLIM1	Hs.368525	NM_020992	9124	1	TC10001549.hg.1	1	NM_020992
PNKP	Hs.78016	NM_007254	11284	1	TC19001727.hg.1	1	NM_007254
PRDX1	Hs.180909	NM_002574	5052	1	TC01002611.hg.1	2	NM_002574; NM_181697
PRDX2	Hs.432121	NM_005809	7001	1	TC19001210.hg.1	4	BC003022; BC064138; NM_005809; NM_181738
PRDX3	Hs.523302	NM_006793	10935	1	TC10001702.hg.1	2	NM_006793; NM_014098
PRDX4	Hs.83383	NM_006406	10549	1	TC0X000110.hg.1	4	BC003609; BC007107; BC016770; NM_006406
PRDX5	Hs.502823	NM_181652	25824	1	TC11000597.hg.1	2	NM_012094; NM_181651
PRDX6	Hs.120	NM_004905	9588	1	TC01001510.hg.1	1	NM_004905
PREX1	Hs.153310	NM_020820	57580	1	TC20000920.hg.1	1	NM_020820
PRNP	Hs.472010	NM_183079	5621	1	TC20000051.hg.1	1	NM_000311
PTGS1	Hs.201978	NM_000962	5742	1	TC09000614.hg.1	3	BC029840; DQ180741; NM_000962
PTGS2	Hs.196384	NM_000963	5743	1	TC01003638.hg.1	4	AJ634912; AY151286; BC013734; NM_000963
PXDN	Hs.332197	NM_012293	7837	1	TC02001512.hg.1	4	XM_056455; XM_935180; XM_935181; XM_935183
RNF7	Hs.134623	NM_014245	9616	1	TC03000764.hg.1	4	AF312226; BC005966; NM_014245; NM_183063
SCARA3	Hs.128856	NM_182826	51435	1	TC08000209.hg.1	3	BC075067; NM_016240; NM_182826
SELS	Hs.32148	NM_203472	55829	1	TC20000544.hg.1	2	NM_018445; NM_203472
SEPP1	Hs.275775	NM_005410	6414	1	TC15002006.hg.1	2	BC058919; NM_005410
SFTPD	Hs.253495	NM_003019	6441	1	TC05001310.hg.1	1	NM_003019
SIRT2	Hs.466693	NM_012237	22933	1	TC10001458.hg.1	3	AK131390; CR617711; NM_012237
SOD1	Hs.443914	NM_000454	6647	1	TC19001508.hg.1	2	BC001034; NM_000454
SOD2	Hs.487046	NM_000636	6648	2	TC21000111.hg.1	3	BC016934; NM_000636; NM_001024465
SOD3	Hs.2420	NM_003102	6649	1	TC06004141.hg.1	2	BC014418; NM_003102
SQSTM1	Hs.437277	NM_003900	8878	1	TC04000173.hg.1	2	BC001874; NM_003900
SRXN1	Hs.516830	NM_080725	140809	1	TC05001053.hg.1	1	NM_080725
STK25	Hs.516807	NM_006374	10494	1	TC02002951.hg.1	2	BC015793; NM_006374

TPO	Hs.467554	NM_000547	7173	1	TC02000013.hg.1	3	NM_000547; NM_175719; NM_175720
TTN	Hs.134602	NM_003319	7273	1	TC02005047.hg.1	3	BC013396; NM_003319; NM_133379
TXN	Hs.435136	NM_003329	7295	1	TC09001473.hg.1	3	BC003377; BX647247; NM_003329
TXNRD1	Hs.728817	NM_003330	7296	1	TC12000810.hg.1	1	NM_003330
TXNRD2	Hs.443430	NM_006440	10587	1	TC22000502.hg.1	3	AF201385; BX957216; NM_006440
UCP2	Hs.80658	NM_003355	7351	1	TC11002074.hg.1	1	NM_003355

Supplementary Table 2. Expression fold changes of 86 genes represented by 166 transcript clusters belonging to oxidative stress related pathways analyzed in U937 cells exposed to different gravitational conditions during the sounding rocket experiment TEXUS-49. Analysis was made with NimbleGen expression microarray.

		**µg vs BL-TX hyp-g**		**BL-TX hyp-g vs H/W 1g GC**		**µg vs H/W 1g GC**	
**gene symbol**	**probeset ID**	**p-value**	**FC**	**p-value**	**FC**	**p-value**	**FC**
ALB	BC034023	0.665	-1.054	0.981	-1.003	0.600	-1.057
ALB	BC035969	0.258	-1.065	0.699	1.029	0.647	-1.035
ALB	BC041789	0.494	1.074	0.090	-1.310	0.050	-1.219
ALB	NM_000477	0.114	1.218	0.143	-1.130	0.475	1.078
ALOX12	BC069557	0.491	-1.130	0.795	1.087	0.891	-1.039
ALOX12	NM_000697	0.004	1.537	0.014	-1.602	0.707	-1.042
AOX1	NM_001159	0.089	-1.250	0.101	1.251	0.992	1.001
APOE	NM_000041	0.284	1.388	0.940	-1.016	0.260	1.367
ATOX1	BC112248	0.163	-1.170	0.796	1.027	0.247	-1.140
ATOX1	NM_004045	0.193	-1.160	0.078	-1.212	0.015	-1.406
BNIP3	BC021989	0.064	-1.246	0.005	1.273	0.855	1.022
BNIP3	NM_004052	0.291	-1.246	0.066	1.490	0.255	1.196
CAT	BC112217	0.080	-1.369	0.001	1.530	0.571	1.118
CAT	NM_001752	0.064	-1.260	0.001	1.378	0.506	1.093
CCL5	BC008600	0.535	1.029	0.590	-1.038	0.861	-1.010
CCL5	NM_002985	0.464	-1.034	0.492	1.043	0.847	1.009
CCS	BC105016	0.552	-1.172	0.192	-1.357	0.056	-1.590
CCS	NM_005125	0.499	-1.109	0.067	-1.346	0.017	-1.493

CYBA	BC006465	0.691	1.027	0.608	-1.053	0.762	-1.026
CYBA	NM_000101	0.244	1.061	0.335	-1.055	0.896	1.006
CYBB	BC032720	0.060	-1.972	0.009	1.819	0.850	-1.084
CYBB	NM_000397	0.077	-1.957	0.010	1.794	0.841	-1.091
CYGB	NM_134268	0.225	1.860	0.954	-1.015	0.189	1.833
DHCR24	BC004375	0.114	-1.163	0.932	1.007	0.176	-1.154
DHCR24	NM_014762	0.181	-1.161	0.918	1.010	0.206	-1.150
DUOX1	NM_017434	0.075	3.304	0.287	-1.891	0.253	1.747
DUOX2	AF230496	0.115	2.070	0.320	-1.444	0.312	1.434
DUOX2	NM_014080	0.130	3.682	0.313	-2.961	0.727	1.244
DUSP1	NM_004417	0.338	-1.366	0.856	1.050	0.432	-1.300
EPHX2	NM_001979	0.186	2.134	0.241	-2.713	0.655	-1.272
EPX	NM_000502	0.800	-1.131	0.656	1.279	0.499	1.131
FOXM1	BC006192	0.309	-1.133	0.472	1.118	0.931	-1.013
FOXM1	NM_021953	0.279	-1.143	0.605	1.078	0.683	-1.060
FOXM1	NM_202003	0.206	-1.161	0.481	1.085	0.570	-1.070
FTH1	BC000857	0.181	-1.140	0.148	1.235	0.446	1.084
FTH1	BC066341	0.637	-1.046	0.203	1.241	0.152	1.187
FTH1	NM_002032	0.112	-1.144	0.053	1.330	0.098	1.162
GCLC	BC039894	0.112	-1.995	0.004	3.699	0.344	1.855
GCLC	NM_001498	0.096	-1.999	0.009	3.329	0.366	1.665
GCLM	BC041809	0.046	-2.118	0.001	2.711	0.633	1.280
GCLM	NM_002061	0.065	-2.094	0.000	4.220	0.277	2.015
GPX1	BC000742	0.259	-1.054	0.122	1.057	0.939	1.003
GPX1	NM_000581	0.150	-1.055	0.451	1.037	0.627	-1.018
GPX1	NM_201397	0.253	-1.052	0.158	1.064	0.794	1.011
GPX2	BC005277	0.126	2.416	0.116	-1.865	0.522	1.295
GPX2	NM_002083	0.099	1.473	0.061	-1.477	0.987	-1.003
GPX3	BC050378	0.317	1.770	0.165	-1.273	0.478	1.391
GPX3	NM_002084	0.533	1.175	0.761	-1.041	0.588	1.128

GPX4	NM_001039847	0.281	1.081	0.010	1.313	0.003	1.419
GPX4	NM_001039848	0.335	1.064	0.014	1.265	0.001	1.346
GPX5	NM_001509	0.276	1.157	0.291	-1.295	0.575	-1.119
GPX5	NM_003996	0.383	1.132	0.016	-1.741	0.017	-1.538
GPX6	NM_182701	0.141	2.515	0.291	-1.886	0.566	1.334
GPX7	BC032788	0.001	1.414	0.155	-1.333	0.668	1.061
GPX7	NM_015696	0.354	1.149	0.559	-1.097	0.746	1.047
GSR	BC069244	0.043	-1.394	0.004	1.632	0.356	1.170
GSR	NM_000637	0.047	-1.625	0.007	1.831	0.655	1.127
GSS	NM_000178	0.695	1.044	0.005	-1.296	0.018	-1.242
GSTP1	BC010915	0.118	-1.058	0.385	1.021	0.245	-1.037
GSTP1	NM_000852	0.833	-1.009	0.919	-1.005	0.733	-1.014
GSTZ1	BC001453	0.071	-1.221	0.374	-1.108	0.032	-1.352
GSTZ1	NM_001513	0.204	-1.127	0.132	-1.138	0.017	-1.282
GSTZ1	NM_145870	0.162	-1.202	0.523	-1.089	0.105	-1.308
GTF2I	BC004472	0.138	-1.670	0.123	1.481	0.743	-1.128
GTF2I	NM_001518	0.059	-1.378	0.001	1.643	0.306	1.192
HMOX1	BC001491	0.151	-1.360	0.973	-1.009	0.265	-1.372
HMOX1	NM_002133	0.221	-1.357	0.677	-1.115	0.173	-1.513
HSPA1A	AK096017	0.115	-1.263	0.020	1.439	0.342	1.139
HSPA1A	BC009322	0.087	-1.536	0.067	1.521	0.972	-1.010
HSPA1A	NM_005345	0.168	-1.208	0.128	1.250	0.792	1.035
KRT1	BC063697	0.075	1.642	0.039	-1.529	0.717	1.074
KRT1	NM_006121	0.140	2.609	0.418	-1.769	0.457	1.475
LOC653361	XM_928908	0.045	1.727	0.084	-1.357	0.273	1.273
LPO	BC107166	0.125	1.691	0.093	-1.388	0.468	1.219
LPO	NM_006151	0.750	1.048	0.869	-1.030	0.912	1.017
MB	BC018001	0.188	2.150	0.294	-1.560	0.438	1.378
MB	NM_005368	0.075	1.826	0.075	-1.666	0.727	1.096
MB	NM_203377	0.757	1.116	0.790	-1.113	0.992	1.003

MB	NM_203378	0.114	1.922	0.043	-1.933	0.985	-1.006
MBL2	BC069338	0.406	1.226	0.438	-1.210	0.948	1.013
MBL2	NM_000242	0.225	1.361	0.184	-1.117	0.348	1.219
MGST3	CR607567	0.856	-1.022	0.061	1.367	0.016	1.338
MGST3	NM_004528	0.671	-1.068	0.129	1.349	0.024	1.263
MPO	NM_000250	0.301	1.296	0.111	-1.212	0.751	1.069
MPO	X04876	0.062	2.308	0.083	-1.547	0.237	1.492
MPV17	NM_002437	0.232	1.062	0.035	1.126	0.001	1.196
MSRA	AY958431	0.034	-1.535	0.002	1.517	0.958	-1.012
MSRA	BC054033	0.036	-1.493	0.008	1.474	0.951	-1.013
MSRA	NM_012331	0.020	-1.555	0.003	1.526	0.924	-1.019
MT3	BC013081	0.043	1.808	0.002	-1.789	0.958	1.010
MT3	NM_005954	0.527	-1.101	0.873	1.027	0.700	-1.072
NCF1	NM_000265	0.037	1.328	0.056	-1.249	0.591	1.064
NCF2	BC001606	0.084	-1.530	0.811	-1.052	0.103	-1.610
NCF2	NM_000433	0.283	-1.306	0.471	-1.190	0.079	-1.554
NCF4	NM_000631	0.143	-1.249	0.078	-1.238	0.009	-1.546
NOS2A	NM_000625	0.132	2.274	0.157	-1.345	0.224	1.690
NOX4	AJ704727	0.204	1.204	0.092	-1.289	0.608	-1.071
NOX4	BC040105	0.172	1.307	0.198	-1.169	0.526	1.118
NOX4	BC051371	0.482	-1.075	0.444	-1.126	0.165	-1.210
NOX4	NM_016931	0.758	1.012	0.255	-1.117	0.246	-1.103
NOX5	AK074071	0.122	2.715	0.389	-1.661	0.324	1.635
NOX5	NM_024505	0.905	-1.035	0.370	1.350	0.281	1.304
NQO1	BC007659	0.063	-1.222	0.020	1.267	0.757	1.037
NQO1	NM_000903	0.054	-1.980	0.000	3.973	0.234	2.007
NUDT1	BC014618	0.295	1.122	0.037	-1.216	0.436	-1.084
NUDT1	BC065367	0.665	1.050	0.001	-1.222	0.133	-1.165
NUDT1	NM_002452	0.883	1.017	0.038	-1.226	0.126	-1.205
NUDT1	NM_198948	0.514	1.062	0.009	-1.209	0.144	-1.138

NUDT1	NM_198949	0.433	1.107	0.021	-1.261	0.285	-1.139
NUDT1	NM_198953	0.350	1.132	0.049	-1.210	0.579	-1.069
OXR1	AB075503	0.584	-1.167	0.055	1.756	0.111	1.505
OXR1	AF309387	0.613	-1.067	0.993	1.002	0.784	-1.064
OXR1	AK096148	0.507	1.042	0.252	-1.109	0.343	-1.064
OXR1	BC032710	0.054	-1.657	0.003	2.214	0.262	1.336
OXR1	NM_181354	0.144	-1.258	0.056	1.317	0.651	1.047
OXSR1	BC008726	0.034	-1.701	0.005	1.530	0.708	-1.111
OXSR1	NM_005109	0.051	-1.587	0.007	1.496	0.824	-1.060
PDLIM1	NM_020992	0.103	2.648	0.201	-2.811	0.904	-1.062
PNKP	NM_007254	0.262	1.146	0.006	-1.343	0.123	-1.172
PRDX1	NM_002574	0.948	-1.005	0.976	-1.003	0.921	-1.008
PRDX1	NM_181697	0.083	-1.153	0.558	-1.048	0.020	-1.208
PRDX2	BC003022	0.033	1.062	0.805	1.011	0.075	1.074
PRDX2	BC064138	0.051	1.137	0.228	-1.071	0.312	1.061
PRDX2	NM_005809	0.148	1.079	0.743	-1.032	0.588	1.045
PRDX2	NM_181738	0.462	1.040	0.458	1.047	0.159	1.089
PRDX3	NM_006793	0.026	-1.291	0.003	1.459	0.301	1.130
PRDX3	NM_014098	0.055	-1.381	0.009	1.616	0.228	1.170
PRDX4	BC003609	0.017	-1.417	0.002	1.567	0.435	1.106
PRDX4	BC007107	0.019	-1.468	0.001	1.611	0.542	1.097
PRDX4	BC016770	0.033	-1.419	0.003	1.590	0.451	1.120
PRDX4	NM_006406	0.042	-1.356	0.001	1.545	0.345	1.140
PRDX5	NM_012094	0.757	1.007	0.001	-1.104	0.003	-1.096
PRDX5	NM_181651	0.197	1.094	0.558	-1.049	0.574	1.043
PRDX6	NM_004905	0.496	-1.062	0.031	1.248	0.153	1.175
PREX1	NM_020820	0.142	-1.219	0.034	1.359	0.460	1.116
PRNP	NM_000311	0.013	-2.237	0.000	2.233	0.997	-1.001
PTGS1	BC029840	0.209	-1.226	0.559	1.092	0.469	-1.123
PTGS1	DQ180741	0.257	-1.230	0.713	1.061	0.402	-1.159

PTGS1	NM_000962	0.052	-1.367	0.005	1.356	0.963	-1.008
PTGS2	AJ634912	0.110	-1.746	0.009	2.253	0.481	1.291
PTGS2	AY151286	0.149	-1.147	0.015	1.223	0.456	1.066
PTGS2	BC013734	0.202	-1.301	0.029	1.520	0.387	1.168
PTGS2	NM_000963	0.008	-1.482	0.003	1.556	0.679	1.050
PXDN	XM_056455	0.101	2.370	0.245	-2.258	0.917	1.049
PXDN	XM_935180	0.098	4.521	0.267	-4.316	0.944	1.047
PXDN	XM_935181	0.333	1.071	0.123	-1.145	0.426	-1.069
PXDN	XM_935183	0.040	2.630	0.178	-2.767	0.910	-1.052
RNF7	AF312226	0.088	-1.419	0.305	1.184	0.237	-1.198
RNF7	BC005966	0.067	-1.447	0.253	1.219	0.248	-1.187
RNF7	NM_014245	0.086	-1.407	0.239	1.233	0.347	-1.141
RNF7	NM_183063	0.055	-1.453	0.182	1.234	0.276	-1.177
SCARA3	BC075067	0.091	2.972	0.187	-2.763	0.882	1.076
SCARA3	NM_016240	0.081	3.008	0.204	-1.771	0.248	1.699
SCARA3	NM_182826	0.165	1.344	0.090	-1.197	0.503	1.123
SELS	NM_018445	0.177	-1.305	0.070	1.501	0.309	1.150
SELS	NM_203472	0.041	-1.856	0.016	1.731	0.849	-1.072
SEPP1	BC058919	0.098	-1.541	0.212	1.361	0.618	-1.132
SEPP1	NM_005410	0.676	-1.184	0.325	1.302	0.820	1.100
SFTPD	NM_003019	0.150	-1.519	0.480	1.263	0.477	-1.203
SIRT2	AK131390	0.757	-1.024	0.225	-1.131	0.145	-1.158
SIRT2	CR617711	0.176	-1.143	0.416	-1.068	0.037	-1.220
SIRT2	NM_012237	0.730	-1.028	0.164	-1.125	0.078	-1.156
SOD1	BC001034	0.907	1.014	0.300	1.171	0.077	1.188
SOD1	NM_000454	0.016	-2.099	0.000	2.429	0.701	1.157
SOD2	BC016934	0.513	-1.062	0.108	-1.552	0.035	-1.648
SOD2	NM_000636	0.059	-2.408	0.021	2.517	0.933	1.045
SOD2	NM_001024465	0.014	-1.547	0.130	1.187	0.087	-1.303
SOD3	BC014418	0.266	1.911	0.482	-1.487	0.543	1.285

SOD3	NM_003102	0.024	1.645	0.111	-1.553	0.775	1.059
SQSTM1	BC001874	0.494	-1.031	0.822	1.014	0.775	-1.016
SQSTM1	NM_003900	0.277	1.158	0.676	-1.049	0.467	1.105
SRXN1	NM_080725	0.148	-1.380	0.714	-1.067	0.077	-1.471
STK25	BC015793	0.190	1.129	0.860	-1.025	0.318	1.102
STK25	NM_006374	0.281	1.076	0.210	1.116	0.059	1.201
TPO	NM_000547	0.110	3.282	0.270	-4.734	0.622	-1.442
TPO	NM_175719	0.091	3.939	0.188	-3.875	0.977	1.016
TPO	NM_175720	0.109	2.697	0.249	-3.815	0.598	-1.415
TTN	BC013396	0.109	3.147	0.241	-2.377	0.585	1.324
TTN	NM_003319	0.176	-1.281	0.399	1.172	0.456	-1.093
TTN	NM_133379	0.307	1.104	0.076	-1.925	0.058	-1.743
TXN	BC003377	0.427	-1.194	0.632	1.152	0.837	-1.036
TXN	BX647247	0.088	-1.646	0.295	1.319	0.146	-1.248
TXN	NM_003329	0.384	-1.210	0.595	1.166	0.825	-1.037
TXNRD1	NM_003330	0.102	-1.888	0.006	2.245	0.735	1.189
TXNRD2	AF201385	0.203	2.342	0.945	-1.013	0.166	2.312
TXNRD2	BX957216	0.368	1.257	0.769	-1.072	0.557	1.173
TXNRD2	NM_006440	0.642	1.046	0.237	-1.131	0.329	-1.081
UCP2	NM_003355	0.523	-1.037	0.661	-1.032	0.266	-1.070

Supplementary Table 3. Expression fold changes of 86 genes represented by 166 transcript clusters belonging to oxidative stress related pathways analyzed in U937 cells exposed to different gravitational conditions during parabolic flight (19th DLR PFC). Analysis was made with NimbleGen expression microarray.

		**1g IF vs H/W 1g GC**		**BL-PFC hyp-g vs 1g IF**		**µg vs BL-PFC hyp-g**		**µg vs 1g IF**		**BL-PFC hyp-g vs H/W 1g GC**		**µg vs H/W 1g GC**	
**gene symbol**	**probeset ID**	**p-value**	**FC**	**p-value**	**FC**	**p-value**	**FC**	**p-value**	**FC**	**p-value**	**FC**	**p-value**	**FC**
ALB	BC034023	0.211	-1.065	0.366	1.031	0.245	-1.030	0.968	1.001	0.543	-1.033	0.170	-1.064
ALB	BC035969	0.355	-1.043	0.992	-1.000	0.534	-1.022	0.465	-1.022	0.424	-1.043	0.157	-1.066
ALB	BC041789	0.603	-1.064	0.436	1.091	0.425	-1.055	0.711	1.034	0.803	1.025	0.713	-1.029
ALB	NM_000477	0.967	-1.002	0.833	-1.006	0.516	1.012	0.831	1.006	0.815	-1.008	0.892	1.004
ALOX12	BC069557	0.956	-1.009	0.063	-1.371	0.287	1.138	0.120	-1.205	0.127	-1.384	0.202	-1.217
ALOX12	NM_000697	0.150	1.114	0.864	1.029	0.378	-1.179	0.126	-1.145	0.495	1.147	0.745	-1.028
AOX1	NM_001159	0.374	1.126	0.861	1.029	0.483	-1.150	0.500	-1.117	0.388	1.159	0.964	1.008
APOE	NM_000041	0.195	-1.212	0.570	1.085	0.996	1.001	0.480	1.086	0.511	-1.117	0.423	-1.117
ATOX1	BC112248	0.362	1.094	0.408	-1.127	0.636	1.074	0.610	-1.050	0.852	-1.031	0.694	1.042
ATOX1	NM_004045	0.626	1.053	0.737	-1.041	0.502	-1.085	0.289	-1.130	0.913	1.012	0.513	-1.072
BNIP3	BC021989	0.886	1.019	0.727	-1.053	0.874	-1.022	0.563	-1.076	0.819	-1.033	0.652	-1.056
BNIP3	NM_004052	0.098	1.228	0.194	-1.146	0.745	-1.040	0.054	-1.192	0.670	1.072	0.826	1.031
CAT	BC112217	0.002	-1.323	0.021	-1.220	0.186	1.140	0.335	-1.070	0.001	-1.614	0.001	-1.415
CAT	NM_001752	0.001	-1.241	0.013	-1.218	0.104	1.148	0.207	-1.061	0.001	-1.511	0.000	-1.317
CCL5	BC008600	0.008	1.157	0.052	-1.119	0.464	1.049	0.201	-1.067	0.598	1.034	0.185	1.084
CCL5	NM_002985	0.011	1.208	0.006	-1.180	0.220	1.077	0.095	-1.096	0.740	1.024	0.187	1.103
CCS	BC105016	0.690	-1.079	0.163	-1.391	0.382	1.312	0.786	-1.060	0.162	-1.501	0.599	-1.144
CCS	NM_005125	0.735	-1.051	0.589	-1.107	0.799	1.068	0.866	-1.036	0.402	-1.163	0.699	-1.089
CYBA	BC006465	0.135	1.161	0.009	-1.382	0.016	1.338	0.574	-1.033	0.288	-1.190	0.228	1.124

CYBA	NM_000101	0.061	1.195	0.007	-1.411	0.037	1.284	0.095	-1.099	0.301	-1.181	0.343	1.087
CYBB	BC032720	0.981	-1.003	0.004	-1.819	0.129	1.366	0.034	-1.332	0.017	-1.825	0.075	-1.336
CYBB	NM_000397	0.948	1.009	0.004	-1.808	0.159	1.362	0.057	-1.328	0.011	-1.793	0.102	-1.316
CYGB	NM_134268	0.240	1.238	0.906	1.019	0.701	-1.047	0.845	-1.027	0.153	1.262	0.152	1.205
DHCR24	BC004375	0.443	1.044	0.284	-1.084	0.550	1.047	0.232	-1.035	0.718	-1.038	0.892	1.008
DHCR24	NM_014762	0.284	1.094	0.040	-1.158	0.350	1.065	0.056	-1.087	0.621	-1.058	0.943	1.006
DUOX1	NM_017434	0.464	-1.104	0.060	1.634	0.211	-1.437	0.562	1.138	0.157	1.480	0.900	1.030
DUOX2	AF230496	0.120	1.388	0.313	1.250	0.662	-1.107	0.503	1.130	0.075	1.736	0.074	1.569
DUOX2	NM_014080	0.261	1.260	0.075	1.514	0.072	-1.479	0.868	1.024	0.037	1.908	0.153	1.290
DUSP1	NM_004417	0.560	1.174	0.533	1.321	0.492	-1.430	0.829	-1.082	0.406	1.552	0.841	1.085
EPHX2	NM_001979	0.377	1.204	0.007	1.941	0.213	-1.384	0.181	1.402	0.011	2.338	0.125	1.689
EPX	NM_000502	0.404	1.287	0.120	1.725	0.344	-1.334	0.299	1.293	0.069	2.219	0.076	1.664
FOXM1	BC006192	0.056	-1.169	0.025	-1.339	0.228	1.161	0.038	-1.153	0.006	-1.564	0.002	-1.348
FOXM1	NM_021953	0.051	-1.186	0.016	-1.363	0.177	1.161	0.033	-1.174	0.002	-1.617	0.001	-1.393
FOXM1	NM_202003	0.085	-1.152	0.015	-1.333	0.174	1.139	0.033	-1.171	0.002	-1.536	0.000	-1.348
FTH1	BC000857	0.935	-1.007	0.024	1.260	0.185	-1.178	0.556	1.070	0.034	1.251	0.628	1.062
FTH1	BC066341	0.888	1.012	0.014	1.267	0.170	-1.123	0.126	1.128	0.019	1.282	0.125	1.141
FTH1	NM_002032	0.837	-1.020	0.030	1.245	0.275	-1.123	0.304	1.109	0.071	1.221	0.456	1.087
GCLC	BC039894	0.128	-1.644	0.252	-1.523	0.646	1.230	0.546	-1.239	0.039	-2.505	0.069	-2.037
GCLC	NM_001498	0.144	-1.526	0.232	-1.405	0.388	1.348	0.880	-1.042	0.048	-2.144	0.153	-1.590
GCLM	BC041809	0.818	-1.043	0.253	-1.304	0.848	1.058	0.279	-1.233	0.300	-1.360	0.306	-1.285
GCLM	NM_002061	0.325	1.269	0.043	-1.810	0.275	1.418	0.280	-1.277	0.291	-1.427	0.982	-1.006
GPX1	BC000742	0.057	-1.077	0.236	1.043	0.391	-1.057	0.813	-1.014	0.345	-1.033	0.186	-1.093
GPX1	NM_000581	0.051	-1.152	0.365	1.064	0.551	-1.054	0.901	1.010	0.316	-1.082	0.142	-1.141
GPX1	NM_201397	0.071	-1.143	0.171	1.099	0.523	-1.053	0.588	1.044	0.565	-1.039	0.280	-1.095
GPX2	BC005277	0.457	1.202	0.124	1.562	0.427	-1.253	0.329	1.247	0.095	1.878	0.167	1.498
GPX2	NM_002083	0.343	1.161	0.060	1.766	0.373	-1.319	0.233	1.339	0.060	2.051	0.150	1.555
GPX3	BC050378	0.330	1.213	0.304	1.232	0.138	-1.323	0.691	-1.074	0.058	1.495	0.456	1.130
GPX3	NM_002084	0.991	-1.002	0.548	1.136	0.485	-1.143	0.971	-1.006	0.501	1.134	0.948	-1.008
GPX4	NM_001039847	0.251	1.044	0.338	-1.055	0.766	1.018	0.429	-1.036	0.849	-1.010	0.862	1.007

GPX4	NM_001039848	0.026	1.112	0.325	-1.037	0.912	1.006	0.529	-1.031	0.154	1.072	0.222	1.079
GPX5	NM_001509	0.334	1.168	0.068	1.451	0.293	-1.243	0.346	1.168	0.041	1.694	0.128	1.363
GPX5	NM_003996	0.187	1.147	0.013	1.494	0.103	-1.285	0.137	1.162	0.011	1.714	0.035	1.334
GPX6	NM_182701	0.246	1.245	0.033	1.666	0.321	-1.296	0.273	1.286	0.023	2.074	0.122	1.601
GPX7	BC032788	0.206	1.313	0.203	1.288	0.647	-1.095	0.382	1.177	0.039	1.691	0.066	1.545
GPX7	NM_015696	0.491	1.164	0.023	1.752	0.210	-1.375	0.282	1.274	0.028	2.039	0.181	1.483
GSR	BC069244	0.822	1.022	0.002	-1.527	0.084	1.283	0.044	-1.190	0.027	-1.494	0.212	-1.164
GSR	NM_000637	0.426	1.093	0.004	-1.542	0.140	1.228	0.013	-1.256	0.067	-1.412	0.240	-1.150
GSS	NM_000178	0.022	-1.108	0.006	-1.204	0.036	1.137	0.217	-1.059	0.000	-1.334	0.001	-1.173
GSTP1	BC010915	0.002	1.263	0.014	-1.217	0.012	1.168	0.366	-1.042	0.630	1.038	0.001	1.212
GSTP1	NM_000852	0.002	1.164	0.008	-1.179	0.023	1.198	0.736	1.016	0.858	-1.013	0.017	1.183
GSTZ1	BC001453	0.001	-1.462	0.182	-1.307	0.454	1.221	0.705	-1.070	0.002	-1.911	0.012	-1.565
GSTZ1	NM_001513	0.000	-1.319	0.138	-1.162	0.834	1.030	0.300	-1.128	0.000	-1.532	0.002	-1.487
GSTZ1	NM_145870	0.016	-1.461	0.130	-1.450	0.373	1.307	0.592	-1.109	0.006	-2.118	0.019	-1.621
GTF2I	BC004472	0.526	-1.095	0.001	-2.014	0.080	1.529	0.010	-1.317	0.016	-2.204	0.057	-1.441
GTF2I	NM_001518	0.925	1.008	0.106	-1.117	0.931	1.007	0.122	-1.109	0.330	-1.108	0.324	-1.100
HMOX1	BC001491	0.238	1.621	0.108	1.877	0.599	-1.247	0.358	1.505	0.031	3.042	0.139	2.439
HMOX1	NM_002133	0.199	1.718	0.224	1.627	0.723	-1.168	0.446	1.393	0.057	2.795	0.147	2.393
HSPA1A	AK096017	0.007	1.590	0.032	1.301	0.126	-1.116	0.141	1.166	0.000	2.069	0.000	1.854
HSPA1A	BC009322	0.006	1.823	0.058	1.346	0.073	-1.198	0.327	1.124	0.000	2.454	0.000	2.049
HSPA1A	NM_005345	0.002	1.493	0.133	1.137	0.383	-1.040	0.211	1.093	0.000	1.698	0.000	1.633
KRT1	BC063697	0.591	1.193	0.046	2.077	0.606	-1.169	0.058	1.777	0.046	2.478	0.044	2.120
KRT1	NM_006121	0.843	1.038	0.020	1.835	0.061	-1.570	0.267	1.169	0.047	1.905	0.328	1.214
LOC653361	XM_928908	0.961	-1.009	0.036	1.499	0.141	-1.272	0.270	1.178	0.071	1.485	0.355	1.167
LPO	BC107166	0.370	-1.118	0.067	1.262	0.526	-1.074	0.115	1.175	0.397	1.129	0.673	1.050
LPO	NM_006151	0.064	-1.137	0.020	1.125	0.102	-1.100	0.628	1.023	0.891	-1.010	0.157	-1.112
MB	BC018001	0.521	1.316	0.146	1.783	0.547	-1.286	0.408	1.386	0.119	2.346	0.266	1.824
MB	NM_005368	0.434	1.378	0.065	2.304	0.167	-1.775	0.391	1.298	0.066	3.175	0.191	1.789
MB	NM_203377	0.228	1.384	0.089	1.968	0.136	-1.774	0.613	1.109	0.070	2.723	0.147	1.535
MB	NM_203378	0.425	1.284	0.105	2.023	0.140	-1.852	0.689	1.092	0.103	2.598	0.288	1.402

MBL2	BC069338	0.259	1.118	0.906	1.011	0.178	1.230	0.134	1.244	0.069	1.130	0.050	1.390
MBL2	NM_000242	0.762	1.015	0.501	1.031	0.144	1.238	0.065	1.276	0.230	1.046	0.090	1.295
MGST3	CR607567	0.673	1.035	0.349	-1.061	0.977	1.002	0.394	-1.058	0.792	-1.025	0.808	-1.022
MGST3	NM_004528	0.311	1.100	0.796	-1.022	0.696	1.038	0.861	1.016	0.427	1.077	0.295	1.118
MPO	NM_000250	0.347	-1.068	0.019	1.148	0.020	-1.117	0.595	1.027	0.265	1.075	0.527	-1.039
MPO	X04876	0.251	1.228	0.820	1.035	0.407	1.156	0.331	1.196	0.052	1.271	0.068	1.469
MPV17	NM_002437	0.025	-1.261	0.078	-1.250	0.590	1.041	0.066	-1.200	0.000	-1.576	0.000	-1.514
MSRA	AY958431	0.550	-1.085	0.701	1.071	0.138	-1.309	0.196	-1.222	0.935	-1.013	0.030	-1.325
MSRA	BC054033	0.492	-1.097	0.653	1.077	0.186	-1.243	0.352	-1.155	0.889	-1.019	0.072	-1.267
MSRA	NM_012331	0.480	-1.094	0.492	1.113	0.198	-1.240	0.496	-1.114	0.888	1.017	0.148	-1.219
MT3	BC013081	0.855	1.030	0.435	1.157	0.452	-1.115	0.776	1.038	0.337	1.192	0.518	1.069
MT3	NM_005954	0.996	-1.001	0.165	1.127	0.182	1.112	0.015	1.254	0.199	1.127	0.027	1.253
NCF1	NM_000265	0.868	-1.028	0.010	1.427	0.034	-1.265	0.172	1.129	0.084	1.389	0.518	1.098
NCF2	BC001606	0.233	-1.201	0.250	-1.265	0.527	1.179	0.692	-1.073	0.088	-1.519	0.218	-1.289
NCF2	NM_000433	0.228	-1.208	0.352	-1.231	0.587	1.172	0.792	-1.051	0.143	-1.487	0.281	-1.269
NCF4	NM_000631	0.120	-1.276	0.029	-1.433	0.074	1.345	0.568	-1.065	0.014	-1.827	0.064	-1.358
NOS2A	NM_000625	0.300	1.085	0.014	1.572	0.139	-1.294	0.053	1.215	0.022	1.706	0.042	1.319
NOX4	AJ704727	0.279	1.122	0.708	1.033	0.973	1.002	0.693	1.035	0.033	1.159	0.074	1.161
NOX4	BC040105	0.332	1.075	0.416	1.071	0.747	-1.028	0.529	1.043	0.162	1.152	0.142	1.121
NOX4	BC051371	0.504	1.094	0.356	-1.132	0.864	1.038	0.659	-1.090	0.752	-1.035	0.989	1.003
NOX4	NM_016931	0.436	1.032	0.058	1.108	0.446	-1.042	0.155	1.064	0.029	1.143	0.053	1.098
NOX5	AK074071	0.735	1.096	0.150	1.528	0.341	-1.317	0.553	1.160	0.139	1.676	0.409	1.272
NOX5	NM_024505	0.969	1.009	0.067	1.794	0.164	-1.489	0.384	1.205	0.093	1.811	0.366	1.216
NQO1	BC007659	0.823	1.019	0.274	-1.125	0.941	1.010	0.216	-1.113	0.509	-1.104	0.468	-1.093
NQO1	NM_000903	0.636	1.128	0.123	-1.532	0.904	1.035	0.116	-1.480	0.337	-1.358	0.347	-1.312
NUDT1	BC014618	0.281	-1.093	0.699	-1.027	0.792	1.023	0.961	-1.004	0.087	-1.123	0.326	-1.098
NUDT1	BC065367	0.239	-1.128	0.932	-1.007	0.912	-1.011	0.864	-1.018	0.159	-1.136	0.219	-1.148
NUDT1	NM_002452	0.128	-1.219	0.921	1.010	0.767	-1.033	0.840	-1.023	0.141	-1.207	0.116	-1.247
NUDT1	NM_198948	0.147	-1.131	0.958	-1.004	0.990	-1.001	0.959	-1.005	0.058	-1.135	0.183	-1.136
NUDT1	NM_198949	0.257	-1.122	0.857	1.015	0.492	-1.068	0.636	-1.052	0.214	-1.105	0.143	-1.180

NUDT1	NM_198953	0.237	-1.138	0.715	1.032	0.817	-1.020	0.907	1.012	0.235	-1.102	0.275	-1.124
OXR1	AB075503	0.032	-1.855	0.800	1.024	0.177	-1.089	0.460	-1.063	0.067	-1.811	0.021	-1.971
OXR1	AF309387	0.889	1.014	0.231	-1.143	0.501	1.067	0.467	-1.072	0.211	-1.128	0.508	-1.057
OXR1	AK096148	0.418	-1.019	0.093	1.033	0.955	1.001	0.097	1.034	0.581	1.013	0.556	1.014
OXR1	BC032710	0.096	-1.228	0.820	-1.015	0.429	-1.032	0.436	-1.048	0.098	-1.247	0.034	-1.287
OXR1	NM_181354	0.214	-1.027	0.997	1.000	0.398	-1.015	0.394	-1.015	0.234	-1.027	0.048	-1.042
OXSR1	BC008726	0.193	-1.090	0.700	-1.030	0.973	1.004	0.785	-1.026	0.222	-1.122	0.309	-1.118
OXSR1	NM_005109	0.136	-1.087	0.103	-1.131	0.735	1.033	0.169	-1.095	0.036	-1.230	0.040	-1.191
PDLIM1	NM_020992	0.374	1.304	0.257	1.339	0.589	-1.144	0.535	1.170	0.071	1.746	0.167	1.526
PNKP	NM_007254	0.477	-1.132	0.579	-1.111	0.792	1.069	0.861	-1.038	0.256	-1.257	0.487	-1.176
PRDX1	NM_002574	0.167	1.058	0.074	-1.085	0.252	1.049	0.309	-1.035	0.589	-1.026	0.553	1.022
PRDX1	NM_181697	0.948	1.002	0.875	-1.005	0.770	-1.011	0.606	-1.017	0.949	-1.003	0.733	-1.014
PRDX2	BC003022	0.013	1.107	0.650	-1.017	0.255	-1.046	0.026	-1.064	0.120	1.089	0.292	1.041
PRDX2	BC064138	0.136	1.134	0.112	1.079	0.129	-1.058	0.646	1.020	0.029	1.223	0.066	1.156
PRDX2	NM_005809	0.007	1.144	0.167	-1.074	0.558	1.028	0.228	-1.045	0.280	1.065	0.032	1.095
PRDX2	NM_181738	0.006	1.231	0.286	-1.077	0.717	-1.020	0.092	-1.100	0.068	1.142	0.031	1.119
PRDX3	NM_006793	0.616	-1.048	0.842	-1.017	0.480	-1.061	0.291	-1.079	0.567	-1.065	0.204	-1.130
PRDX3	NM_014098	0.241	-1.079	0.879	-1.012	0.367	-1.078	0.144	-1.091	0.338	-1.092	0.027	-1.178
PRDX4	BC003609	0.571	1.028	0.772	1.020	0.510	-1.051	0.561	-1.030	0.547	1.049	0.975	-1.002
PRDX4	BC007107	0.574	1.031	0.515	1.055	0.217	-1.112	0.253	-1.054	0.401	1.088	0.697	-1.022
PRDX4	BC016770	0.728	1.019	0.397	1.075	0.384	-1.082	0.897	-1.007	0.380	1.096	0.843	1.012
PRDX4	NM_006406	0.407	1.043	0.624	1.044	0.581	-1.052	0.881	-1.008	0.406	1.088	0.560	1.034
PRDX5	NM_012094	0.002	1.174	0.166	-1.086	0.221	1.077	0.781	-1.008	0.332	1.080	0.004	1.164
PRDX5	NM_181651	0.023	1.114	0.555	-1.037	0.511	1.037	0.995	1.000	0.301	1.074	0.005	1.114
PRDX6	NM_004905	0.738	1.036	0.386	-1.118	0.429	1.093	0.784	-1.023	0.583	-1.080	0.888	1.012
PREX1	NM_020820	0.114	-1.148	0.215	-1.137	0.650	1.056	0.346	-1.077	0.056	-1.305	0.049	-1.237
PRNP	NM_000311	0.462	-1.113	0.397	1.200	0.387	-1.258	0.820	-1.048	0.731	1.078	0.470	-1.167
PTGS1	BC029840	0.004	1.315	0.001	-1.560	0.046	1.275	0.002	-1.224	0.280	-1.186	0.391	1.075
PTGS1	DQ180741	0.008	1.312	0.002	-1.636	0.029	1.356	0.011	-1.206	0.201	-1.247	0.304	1.088
PTGS1	NM_000962	0.000	1.350	0.052	-1.137	0.224	1.065	0.216	-1.068	0.013	1.187	0.000	1.264

PTGS2	AJ634912	0.210	-1.167	0.008	1.414	0.069	-1.253	0.171	1.129	0.215	1.212	0.790	-1.034
PTGS2	AY151286	0.057	-1.059	0.103	1.024	0.144	-1.022	0.894	1.002	0.301	-1.034	0.066	-1.057
PTGS2	BC013734	0.127	-1.054	0.442	1.015	0.583	-1.013	0.917	1.002	0.321	-1.039	0.166	-1.052
PTGS2	NM_000963	0.075	-1.062	0.008	1.088	0.225	-1.038	0.030	1.048	0.580	1.024	0.690	-1.014
PXDN	XM_056455	0.891	1.018	0.006	1.655	0.019	-1.495	0.431	1.107	0.010	1.684	0.363	1.127
PXDN	XM_935180	0.576	1.202	0.023	2.042	0.130	-1.488	0.150	1.372	0.033	2.454	0.107	1.649
PXDN	XM_935181	0.830	1.003	0.097	1.027	0.026	-1.043	0.200	-1.016	0.167	1.030	0.445	-1.013
PXDN	XM_935183	0.989	-1.002	0.012	2.059	0.115	-1.531	0.176	1.345	0.028	2.055	0.229	1.342
RNF7	AF312226	0.715	-1.035	0.039	1.217	0.023	-1.246	0.689	-1.024	0.199	1.175	0.538	-1.060
RNF7	BC005966	0.821	-1.024	0.062	1.193	0.024	-1.247	0.316	-1.045	0.297	1.164	0.524	-1.071
RNF7	NM_014245	0.742	-1.034	0.058	1.191	0.017	-1.268	0.226	-1.064	0.298	1.153	0.361	-1.100
RNF7	NM_183063	0.830	-1.021	0.065	1.197	0.015	-1.272	0.255	-1.062	0.234	1.172	0.389	-1.085
SCARA3	BC075067	0.637	1.136	0.028	2.184	0.113	-1.689	0.308	1.293	0.042	2.481	0.234	1.469
SCARA3	NM_016240	0.394	1.200	0.135	1.353	0.730	1.089	0.101	1.474	0.083	1.624	0.070	1.769
SCARA3	NM_182826	0.309	1.259	0.195	1.389	0.352	-1.280	0.647	1.085	0.124	1.748	0.242	1.366
SELS	NM_018445	0.941	1.009	0.427	1.080	0.062	-1.196	0.063	-1.107	0.587	1.089	0.435	-1.098
SELS	NM_203472	0.120	-1.214	0.753	1.062	0.368	-1.271	0.379	-1.197	0.470	-1.143	0.076	-1.453
SEPP1	BC058919	0.052	-1.285	0.174	1.061	0.347	-1.053	0.855	1.008	0.184	-1.211	0.066	-1.275
SEPP1	NM_005410	0.127	-1.245	0.707	1.027	0.779	-1.018	0.892	1.009	0.220	-1.213	0.132	-1.234
SFTPD	NM_003019	0.961	-1.004	0.493	1.067	0.044	-1.167	0.285	-1.093	0.444	1.063	0.153	-1.098
SIRT2	AK131390	0.804	-1.018	0.136	-1.210	0.501	1.109	0.378	-1.091	0.145	-1.232	0.338	-1.111
SIRT2	CR617711	0.993	1.001	0.009	-1.293	0.120	1.134	0.062	-1.140	0.028	-1.292	0.124	-1.139
SIRT2	NM_012237	0.880	-1.010	0.003	-1.265	0.119	1.128	0.053	-1.122	0.015	-1.278	0.100	-1.133
SOD1	BC001034	0.336	1.084	0.105	-1.102	0.868	1.014	0.239	-1.087	0.861	-1.017	0.973	-1.003
SOD1	NM_000454	0.702	-1.071	0.380	-1.223	0.812	-1.052	0.186	-1.286	0.205	-1.309	0.062	-1.377
SOD2	BC016934	0.103	1.227	0.927	1.018	0.906	1.023	0.688	1.041	0.375	1.249	0.063	1.277
SOD2	NM_000636	0.356	1.222	0.032	-1.946	0.428	1.314	0.082	-1.480	0.188	-1.593	0.455	-1.212
SOD2	NM_001024465	0.000	1.422	0.541	-1.045	0.880	-1.012	0.310	-1.058	0.006	1.360	0.000	1.344
SOD3	BC014418	0.113	1.618	0.075	1.558	0.333	-1.254	0.342	1.242	0.011	2.521	0.031	2.010
SOD3	NM_003102	0.614	1.084	0.048	1.510	0.344	-1.208	0.134	1.251	0.064	1.637	0.119	1.355

SQSTM1	BC001874	0.159	1.120	0.795	-1.021	0.848	1.018	0.966	-1.003	0.330	1.097	0.235	1.116
SQSTM1	NM_003900	0.388	1.110	0.002	-1.503	0.081	1.296	0.123	-1.160	0.112	-1.354	0.761	-1.045
SRXN1	NM_080725	0.097	1.303	0.676	-1.051	0.590	-1.075	0.379	-1.130	0.133	1.239	0.400	1.153
STK25	BC015793	0.555	-1.046	0.272	-1.114	0.017	1.261	0.093	1.132	0.121	-1.165	0.243	1.083
STK25	NM_006374	0.756	-1.019	0.131	-1.168	0.191	1.138	0.689	-1.027	0.082	-1.190	0.411	-1.046
TPO	NM_000547	0.261	1.273	0.097	1.764	0.365	-1.342	0.204	1.315	0.081	2.245	0.087	1.673
TPO	NM_175719	0.138	1.324	0.041	1.602	0.400	-1.215	0.152	1.319	0.020	2.122	0.040	1.747
TPO	NM_175720	0.037	1.153	0.017	1.498	0.058	-1.399	0.455	1.071	0.014	1.728	0.089	1.235
TTN	BC013396	0.231	1.539	0.045	1.822	0.491	-1.187	0.096	1.535	0.015	2.804	0.018	2.362
TTN	NM_003319	0.474	-1.079	0.534	-1.046	0.497	-1.046	0.243	-1.094	0.276	-1.128	0.132	-1.180
TTN	NM_133379	0.494	1.101	0.050	1.769	0.197	-1.440	0.263	1.229	0.061	1.947	0.175	1.353
TXN	BC003377	0.190	1.174	0.668	1.044	0.469	-1.066	0.795	-1.021	0.143	1.226	0.198	1.150
TXN	BX647247	0.123	1.215	0.835	1.023	0.913	-1.010	0.907	1.013	0.031	1.243	0.059	1.230
TXN	NM_003329	0.193	1.144	0.843	1.016	0.740	-1.027	0.891	-1.010	0.187	1.162	0.215	1.132
TXNRD1	NM_003330	0.640	-1.097	0.806	1.050	0.848	-1.051	0.996	-1.001	0.865	-1.044	0.716	-1.098
TXNRD2	AF201385	0.241	-1.248	0.094	-1.466	0.241	1.140	0.169	-1.286	0.002	-1.829	0.003	-1.605
TXNRD2	BX957216	0.731	-1.062	0.023	-1.758	0.338	1.255	0.083	-1.401	0.014	-1.867	0.046	-1.488
TXNRD2	NM_006440	0.286	-1.096	0.012	-1.446	0.261	1.224	0.079	-1.181	0.020	-1.584	0.053	-1.295
UCP2	NM_003355	0.449	1.031	0.017	-1.137	0.489	1.035	0.036	-1.099	0.061	-1.103	0.148	-1.065

Supplementary Table 4. Expression fold changes of 86 genes represented by 106 transcript clusters belonging to oxidative stress related pathways analyzed in Jurkat T cells exposed to different gravitational conditions during the suborbital ballistic rocket experiment TEXUS-51. Analysis was made with the Affymetrix GeneChip® Human Transcriptome Array.

		**H/W 1g GC vs CC**		**BL-TX hyp-g vs H/W 1g GC**		**BL-TX hyp-g vs 1g IF**		**µg vs 1g IF**		**1g IF vs H/W 1g GC**		**µg vs BL-TX hyp-g**	
**gene symbol**	**transcript cluster ID**	**FC**	**p-value**	**FC**	**p-value**	**FC**	**p-value**	**FC**	**p-value**	**FC**	**p-value**	**FC**	**p-value**
ALB	TC04000403.hg.1	1.130	0.000	-1.010	0.010	-1.080	0.000	-1.070	0.001	1.070	0.014	1.000	0.250
ALOX12	TC17000081.hg.1	1.230	0.000	-1.100	0.007	-1.150	0.000	-1.140	0.000	1.040	0.921	1.010	0.920
AOX1	TC02001162.hg.1	1.130	0.000	-1.050	0.018	-1.060	0.001	-1.040	0.005	1.000	0.958	1.020	0.477
APOE	TC19000633.hg.1	1.500	0.000	-1.170	0.003	-1.210	0.000	-1.200	0.003	1.030	0.765	1.010	0.455
ATOX1	TC05001957.hg.1	1.100	0.002	-1.090	0.005	-1.090	0.001	-1.100	0.000	-1.000	0.550	-1.010	0.501
BNIP3	TC10001769.hg.1	-2.630	0.000	1.150	0.002	1.140	0.042	1.150	0.060	1.000	0.201	1.000	0.954
CAT	TC11000327.hg.1	-1.140	0.003	1.140	0.030	1.310	0.000	1.280	0.001	-1.150	0.032	-1.030	0.776
CCL5	TC17001378.hg.1	1.160	0.000	-1.030	0.027	-1.070	0.067	-1.080	0.049	1.040	0.979	-1.000	0.783
CCS	TC11000672.hg.1	1.290	0.000	-1.100	0.004	-1.120	0.008	-1.090	0.041	1.020	0.521	1.020	0.482
CYBA	TC16001339.hg.1	1.270	0.000	-1.030	0.395	-1.170	0.000	-1.150	0.001	1.140	0.000	1.020	0.463
CYBB	TC0X000171.hg.1	1.150	0.001	-1.040	0.530	-1.040	0.044	-1.040	0.005	1.000	0.350	1.000	0.671
CYGB	TC17001902.hg.1	1.510	0.000	-1.160	0.013	-1.170	0.000	-1.180	0.000	1.010	0.754	-1.010	0.924
DHCR24	TC01002694.hg.1	1.010	0.624	-1.040	0.026	-1.030	0.020	-1.020	0.120	-1.010	0.418	1.020	0.185
DUOX1	TC15000352.hg.1	1.320	0.000	-1.140	0.004	-1.150	0.000	-1.170	0.000	1.020	0.458	-1.010	0.803
DUOX2	TC15001305.hg.1	1.340	0.000	-1.130	0.012	-1.130	0.000	-1.130	0.000	1.000	0.510	1.000	0.505
DUSP1	TC05002066.hg.1	-1.100	0.007	-1.060	0.155	-1.060	0.102	-1.130	0.022	-1.000	0.735	-1.060	0.429
EPHX2	TC08000208.hg.1	-1.280	0.000	-1.020	0.428	1.060	0.115	1.100	0.000	-1.080	0.031	1.050	0.048
EPX	TC17000711.hg.1	1.440	0.000	-1.250	0.007	-1.230	0.000	-1.260	0.000	-1.010	0.735	-1.020	0.504
FOXM1	TC12001106.hg.1	-1.120	0.023	1.120	0.197	-1.080	0.015	-1.040	0.161	1.210	0.001	1.040	0.042

FTH1	TC11001860.hg.1	-1.620	0.000	1.150	0.032	1.060	0.234	1.010	0.426	1.080	0.282	-1.050	0.497
GCLC	TC06004051.hg.1	-1.320	0.000	1.230	0.000	1.170	0.001	1.110	0.027	1.050	0.141	-1.050	0.498
GCLM	TC01002882.hg.1	-1.530	0.000	1.340	0.000	1.240	0.003	1.210	0.025	1.080	0.273	-1.020	0.668
GPX1	TC03001407.hg.1	1.490	0.000	1.030	0.398	-1.080	0.007	-1.080	0.000	1.110	0.000	-1.000	0.692
GPX2	TC14001223.hg.1	1.200	0.000	-1.080	0.003	-1.070	0.001	-1.010	0.971	-1.000	0.175	1.060	0.138
GPX3	TC05000837.hg.1	1.380	0.000	-1.120	0.009	-1.100	0.001	-1.100	0.003	-1.020	0.744	-1.000	0.697
GPX4	TC19000030.hg.1	1.040	0.179	1.060	0.656	-1.070	0.031	-1.080	0.045	1.130	0.002	-1.010	0.425
GPX5	TC06000286.hg.1	1.240	0.000	-1.090	0.067	-1.030	0.175	-1.040	0.406	-1.060	0.609	-1.010	0.528
GPX5	TC6_cox_hap2000001.hg.1	1.260	0.004	-1.140	0.082	-1.060	0.204	-1.030	0.868	-1.080	0.432	1.030	0.236
GPX6	TC06001444.hg.1	1.130	0.011	-1.020	0.470	-1.100	0.010	-1.110	0.002	1.080	0.181	-1.000	0.887
GPX6	TC6_cox_hap2000110.hg.1	1.170	0.106	-1.220	0.292	-1.190	0.119	-1.130	0.024	-1.020	0.714	1.060	0.912
GPX7	TC01000643.hg.1	1.280	0.000	-1.140	0.003	-1.160	0.004	-1.200	0.000	1.020	0.513	-1.040	0.243
GSR	TC08001110.hg.1	1.030	0.427	1.100	0.001	1.110	0.010	1.100	0.054	-1.000	0.335	-1.000	0.627
GSS	TC20000789.hg.1	1.210	0.000	-1.000	0.227	-1.060	0.024	1.040	0.396	1.050	0.142	1.090	0.004
GSTP1	TC11000694.hg.1	1.090	0.001	1.000	0.478	-1.030	0.080	-1.010	0.553	1.030	0.127	1.020	0.118
GSTZ1	TC14000489.hg.1	1.420	0.000	-1.070	0.128	-1.130	0.002	-1.060	0.047	1.050	0.004	1.060	0.085
GTF2I	TC07000458.hg.1	-2.770	0.000	1.580	0.000	1.260	0.009	1.210	0.073	1.250	0.004	-1.050	0.578
HMOX1	TC22000259.hg.1	1.230	0.000	-1.120	0.009	-1.180	0.000	-1.240	0.000	1.060	0.557	-1.050	0.086
HSPA1A	TC06001547.hg.1	1.330	0.000	-1.100	0.107	-1.070	0.204	-1.120	0.011	-1.030	0.669	-1.050	0.283
HSPA1A	TC6_apd_hap1000088.hg.1	1.300	0.000	-1.020	0.224	-1.000	0.527	-1.090	0.018	-1.020	0.438	-1.090	0.137
HSPA1A	TC6_cox_hap2000174.hg.1	1.280	0.000	-1.020	0.266	-1.010	0.562	-1.080	0.019	-1.010	0.431	-1.060	0.168
HSPA1A	TC6_dbb_hap3000163.hg.1	1.280	0.000	-1.060	0.243	-1.040	0.433	-1.100	0.014	-1.020	0.636	-1.060	0.146
HSPA1A	TC6_qbl_hap6000164.hg.1	1.320	0.000	-1.050	0.184	-1.060	0.338	-1.090	0.013	1.010	0.541	-1.030	0.225
HSPA1A; HSPA1B	TC06000384.hg.1	-1.070	0.144	-1.050	0.378	1.060	0.954	-1.040	0.231	-1.110	0.187	-1.100	0.346
HSPA1A; HSPA1B	TC6_apd_hap1000039.hg.1	1.020	0.824	-1.060	0.366	1.020	0.957	-1.070	0.192	-1.080	0.241	-1.090	0.329
HSPA1A; HSPA1B	TC6_cox_hap2000078.hg.1	1.020	0.891	-1.060	0.375	1.040	0.985	-1.040	0.274	-1.100	0.232	-1.080	0.377
HSPA1A; HSPA1B	TC6_dbb_hap3000069.hg.1	1.030	0.753	-1.070	0.338	1.040	0.971	-1.050	0.211	-1.110	0.181	-1.090	0.313

HSPA1A; HSPA1B	TC6_qbl_hap6000069.hg.1	1.010	0.985	-1.070	0.343	1.030	0.969	-1.050	0.249	-1.110	0.216	-1.080	0.384
HSPA1B; HSPA1A	TC06000385.hg.1	-1.170	0.014	-1.050	0.601	1.080	0.829	-1.010	0.360	-1.130	0.354	-1.090	0.359
HSPA1B; HSPA1A	TC6_apd_hap1000040.hg.1	-1.290	0.003	-1.020	0.816	1.150	0.607	1.060	0.724	-1.170	0.409	-1.090	0.403
HSPA1B; HSPA1A	TC6_cox_hap2000079.hg.1	-1.340	0.002	1.000	0.937	1.160	0.563	1.070	0.810	-1.160	0.476	-1.090	0.414
HSPA1B; HSPA1A	TC6_dbb_hap3000070.hg.1	-1.330	0.002	-1.010	0.844	1.160	0.585	1.070	0.786	-1.170	0.412	-1.090	0.422
HSPA1B; HSPA1A	TC6_qbl_hap6000070.hg.1	-1.210	0.016	1.010	0.919	1.150	0.616	1.050	0.616	-1.140	0.501	-1.100	0.343
KRT1	TC12001532.hg.1	1.090	0.027	-1.010	0.385	1.000	0.785	-1.000	0.571	-1.020	0.703	-1.010	0.589
LOC100129518; SOD2	TC06004142.hg.1	-1.190	0.010	-1.010	0.371	-1.140	0.010	-1.210	0.011	1.120	0.078	-1.060	0.504
LPO	TC17000712.hg.1	1.240	0.000	-1.130	0.012	-1.150	0.000	-1.140	0.000	1.030	0.977	1.010	0.796
MB	TC22000697.hg.1	1.390	0.000	-1.150	0.005	-1.130	0.000	-1.130	0.006	-1.010	0.898	1.000	0.402
MB; LOC284912	TC22000698.hg.1	1.280	0.001	-1.170	0.011	-1.120	0.075	-1.090	0.142	-1.050	0.460	1.030	0.520
MBL2	TC10001301.hg.1	1.090	0.013	-1.030	0.390	-1.070	0.040	-1.060	0.032	1.040	0.251	1.010	0.665
MGST3	TC01001444.hg.1	-1.200	0.000	-1.020	0.713	1.030	0.387	-1.010	0.844	-1.050	0.736	-1.050	0.331
MGST3; LOC100505828	TC01001443.hg.1	1.130	0.001	1.030	0.263	1.040	0.087	1.100	0.004	-1.000	0.516	1.060	0.068
MPO	TC17001727.hg.1	1.360	0.000	-1.100	0.040	-1.160	0.002	-1.160	0.001	1.050	0.589	-1.000	0.319
MPV17	TC02001681.hg.1	1.010	0.544	-1.030	0.121	-1.030	0.018	-1.010	0.125	-1.010	0.255	1.020	0.295
MSRA	TC08000071.hg.1	1.400	0.000	-1.140	0.006	-1.090	0.057	-1.090	0.041	-1.050	0.581	-1.000	0.706
MSRA	TC08000073.hg.1	1.350	0.000	-1.250	0.004	-1.110	0.032	-1.080	0.020	-1.130	0.035	1.020	0.594
MT3	TC16000465.hg.1	1.350	0.000	-1.090	0.027	-1.100	0.000	-1.130	0.000	1.000	0.983	-1.030	0.531
NCF1	TC07000459.hg.1	1.310	0.000	-1.150	0.001	-1.100	0.000	-1.150	0.000	-1.040	0.376	-1.040	0.247
NCF2	TC01003616.hg.1	1.200	0.000	-1.070	0.010	-1.090	0.001	-1.080	0.003	1.020	0.457	1.010	0.413

NCF4	TC22000270.hg.1	1.370	0.000	-1.160	0.001	-1.120	0.002	-1.110	0.004	-1.040	0.294	1.010	0.804
NOS2	TC17002894.hg.1	1.270	0.000	-1.110	0.001	-1.150	0.000	-1.170	0.000	1.030	0.285	-1.020	0.632
NOX4	TC11002178.hg.1	1.210	0.000	-1.060	0.019	-1.070	0.002	-1.080	0.001	1.010	0.619	-1.010	0.431
NOX5; SPESP1	TC15000630.hg.1	1.240	0.000	-1.080	0.013	-1.120	0.001	-1.130	0.000	1.030	0.398	-1.010	0.503
NQO1	TC16001225.hg.1	1.150	0.001	1.000	0.814	-1.070	0.077	-1.030	0.848	1.070	0.047	1.040	0.076
NUDT1	TC07000029.hg.1	1.530	0.000	-1.110	0.007	-1.200	0.001	-1.130	0.020	1.080	0.149	1.070	0.021
OXR1; RP11-395G23.3	TC08000660.hg.1	-1.060	0.001	1.010	0.448	1.020	0.340	-1.010	0.817	-1.000	0.735	-1.020	0.371
OXSR1	TC03000188.hg.1	-2.420	0.000	1.500	0.001	1.430	0.000	1.320	0.021	1.050	0.540	-1.080	0.484
PDLIM1	TC10001549.hg.1	-1.200	0.007	1.090	0.532	1.010	0.518	1.000	0.873	1.070	0.208	-1.010	0.468
PNKP	TC19001727.hg.1	1.350	0.000	-1.260	0.000	-1.170	0.004	-1.140	0.042	-1.070	0.007	1.030	0.391
PRDX1	TC01002611.hg.1	-1.090	0.039	1.070	0.060	1.170	0.000	1.210	0.004	-1.090	0.083	1.030	0.917
PRDX2	TC19001210.hg.1	-1.000	0.889	1.010	0.388	-1.020	0.151	-1.020	0.401	1.030	0.385	1.000	0.317
PRDX3	TC10001702.hg.1	-1.810	0.000	1.390	0.001	1.370	0.002	1.230	0.051	1.010	0.795	-1.110	0.233
PRDX4	TC0X000110.hg.1	-1.130	0.003	1.190	0.002	1.080	0.304	1.030	0.536	1.100	0.042	-1.050	0.666
PRDX5	TC11000597.hg.1	1.420	0.000	1.080	0.048	1.050	0.452	1.090	0.037	1.030	0.151	1.030	0.222
PRDX6	TC01001510.hg.1	-1.140	0.015	1.150	0.010	1.030	0.322	1.010	0.495	1.120	0.050	-1.020	0.412
PREX1	TC20000920.hg.1	1.170	0.000	-1.010	0.180	-1.040	0.005	-1.010	0.406	1.040	0.198	1.030	0.053
PRNP	TC20000051.hg.1	1.180	0.000	-1.040	0.268	1.070	0.000	1.040	0.003	-1.110	0.000	-1.030	0.104
PTGS1	TC09000614.hg.1	1.280	0.000	-1.080	0.013	-1.110	0.001	-1.110	0.001	1.030	0.635	-1.000	0.976
PTGS2	TC01003638.hg.1	1.190	0.000	-1.060	0.037	-1.090	0.002	-1.090	0.002	1.030	0.201	-1.000	0.743
PXDN	TC02001512.hg.1	1.000	0.476	1.010	0.567	1.030	0.321	1.040	0.030	-1.010	0.683	1.010	0.312
RNF7	TC03000764.hg.1	-1.650	0.000	1.080	0.019	1.100	0.019	1.090	0.132	-1.020	0.892	-1.010	0.371
SCARA3	TC08000209.hg.1	1.300	0.000	-1.140	0.004	-1.150	0.000	-1.130	0.002	1.000	0.972	1.010	0.681
SCRT2; SRXN1; PS1TP5; RP5-850E9.3	TC20000544.hg.1	1.300	0.000	-1.090	0.040	-1.040	0.019	-1.020	0.086	-1.050	0.458	1.020	0.553

SEPP1	TC05001310.hg.1	1.110	0.001	-1.060	0.031	-1.060	0.013	-1.020	0.183	1.000	0.484	1.040	0.229
SFTPD	TC10001458.hg.1	1.420	0.000	-1.190	0.005	-1.170	0.001	-1.160	0.005	-1.020	0.794	1.010	0.835
SIRT2	TC19001508.hg.1	1.220	0.000	-1.200	0.000	-1.120	0.001	-1.120	0.007	-1.070	0.007	1.000	0.702
SOD1	TC21000111.hg.1	-1.420	0.000	1.300	0.001	1.200	0.003	1.270	0.001	1.080	0.156	1.060	0.595
SOD2; LOC100129518	TC06004141.hg.1	-1.060	0.001	1.030	0.168	1.040	0.044	1.040	0.041	-1.020	0.282	-1.000	0.924
SOD3	TC04000173.hg.1	1.400	0.000	-1.160	0.027	-1.200	0.004	-1.210	0.002	1.030	0.390	-1.010	0.831
SQSTM1	TC05001053.hg.1	1.170	0.000	-1.100	0.004	-1.120	0.000	-1.070	0.002	1.010	0.665	1.040	0.118
STK25	TC02002951.hg.1	1.230	0.000	-1.030	0.047	-1.110	0.000	-1.100	0.000	1.080	0.000	1.010	0.257
TPO	TC02000013.hg.1	1.290	0.000	-1.070	0.009	-1.110	0.000	-1.100	0.000	1.040	0.013	1.010	0.265
TTN	TC02005047.hg.1	1.140	0.000	-1.050	0.007	-1.070	0.000	-1.070	0.001	1.010	0.528	1.000	0.844
TXN	TC09001473.hg.1	-1.670	0.000	1.370	0.000	1.290	0.000	1.250	0.004	1.060	0.156	-1.030	0.595
TXNRD1	TC12000810.hg.1	-1.650	0.000	1.370	0.000	1.300	0.001	1.220	0.022	1.060	0.254	-1.060	0.525
TXNRD2	TC22000502.hg.1	1.280	0.000	-1.100	0.006	-1.120	0.000	-1.130	0.000	1.020	0.637	-1.010	0.495
UCP2	TC11002074.hg.1	-1.150	0.001	1.020	0.891	-1.130	0.005	-1.050	0.099	1.150	0.001	1.070	0.114
VIMP	TC15002006.hg.1	-1.010	0.404	1.120	0.002	1.020	0.807	1.050	0.175	1.100	0.004	1.030	0.276

Supplementary Table 5. Expression fold changes of 86 genes represented by 106 transcript clusters belonging to oxidative stress related pathways analyzed in Jurkat T cells exposed to different gravitational conditions during parabolic flight (23rd DLR PFC). Analysis was made with the Affymetrix GeneChip® Human Transcriptome Array.

		1g IF vs H/W 1g GC		BL -PFC hyp-g vs 1g IF		µg vs 1g IF		µg vs BL-PFC hyp-g	
**gene symbol**	**transcript Cluster ID**	**FC**	**p-value**	**FC**	**p-value**	**FC**	**p-value**	**FC**	**p-value**
ALB	TC04000403.hg.1	1.070	0.016	-1.020	0.285	-1.010	0.988	1.010	0.383
ALOX12	TC17000081.hg.1	1.130	0.000	-1.040	0.158	-1.090	0.050	-1.050	0.431
AOX1	TC02001162.hg.1	1.050	0.069	-1.040	0.197	-1.020	0.039	1.020	0.441
APOE	TC19000633.hg.1	1.060	0.024	-1.060	0.138	-1.050	0.257	1.010	0.599
ATOX1	TC05001957.hg.1	-1.000	0.591	-1.060	0.015	-1.010	0.216	1.050	0.106
BNIP3	TC10001769.hg.1	1.040	0.177	1.020	0.140	1.000	0.297	-1.010	0.467
CAT	TC11000327.hg.1	1.020	0.273	1.070	0.014	1.030	0.287	-1.040	0.144
CCL5	TC17001378.hg.1	1.090	0.003	1.000	0.513	1.020	0.131	1.020	0.280
CCS	TC11000672.hg.1	-1.010	0.167	-1.020	0.016	-1.020	0.196	1.010	0.477
CYBA	TC16001339.hg.1	1.090	0.013	-1.010	0.787	-1.000	0.930	1.010	0.702
CYBB	TC0X000171.hg.1	1.050	0.104	-1.060	0.200	-1.130	0.082	-1.060	0.324
CYGB	TC17001902.hg.1	1.090	0.007	-1.010	0.503	-1.030	0.280	-1.020	0.476
DHCR24	TC01002694.hg.1	-1.280	0.000	1.100	0.023	1.040	0.289	-1.060	0.433
DUOX1	TC15000352.hg.1	1.110	0.003	-1.060	0.015	-1.060	0.050	1.000	0.338
DUOX2	TC15001305.hg.1	1.100	0.005	-1.070	0.002	-1.070	0.019	-1.000	0.881
DUSP1	TC05002066.hg.1	-1.030	0.164	-1.060	0.664	-1.040	0.215	1.010	0.436
EPHX2	TC08000208.hg.1	-1.550	0.000	-1.140	0.002	-1.070	0.164	1.070	0.329
EPX	TC17000711.hg.1	1.100	0.007	-1.100	0.008	-1.080	0.059	1.020	0.403
FOXM1	TC12001106.hg.1	-1.120	0.043	1.140	0.241	1.110	0.062	-1.020	0.686
FTH1	TC11001860.hg.1	-1.060	0.025	1.010	0.384	1.010	0.681	-1.000	0.584

GCLC	TC06004051.hg.1	-1.110	0.000	1.020	0.100	1.030	0.065	1.000	0.942
GCLM	TC01002882.hg.1	-1.180	0.000	1.110	0.002	1.090	0.018	-1.020	0.634
GPX1	TC03001407.hg.1	-1.110	0.024	1.020	0.357	1.030	0.745	1.010	0.544
GPX2	TC14001223.hg.1	1.140	0.140	-1.120	0.116	-1.010	0.823	1.110	0.173
GPX3	TC05000837.hg.1	1.100	0.021	-1.030	0.280	-1.100	0.010	-1.060	0.087
GPX4	TC19000030.hg.1	1.040	0.138	-1.030	0.770	-1.040	0.173	-1.010	0.273
GPX5	TC06000286.hg.1	1.050	0.115	-1.020	0.883	-1.030	0.754	-1.010	0.703
GPX5	TC6_cox_hap2000001.hg.1	1.100	0.160	-1.010	0.930	1.010	0.619	1.020	0.619
GPX6	TC06001444.hg.1	1.010	0.700	-1.040	0.605	-1.030	0.476	1.010	0.808
GPX6	TC6_cox_hap2000110.hg.1	-1.040	0.512	-1.000	0.978	-1.130	0.463	-1.130	0.408
GPX7	TC01000643.hg.1	1.000	0.330	-1.020	0.372	1.030	0.976	1.050	0.507
GSR	TC08001110.hg.1	1.100	0.001	1.110	0.001	1.060	0.008	-1.050	0.584
GSS	TC20000789.hg.1	-1.040	0.760	1.000	0.268	-1.050	0.059	-1.050	0.298
GSTP1	TC11000694.hg.1	-1.020	0.543	-1.000	0.867	-1.020	0.277	-1.020	0.083
GSTZ1	TC14000489.hg.1	-1.070	0.033	-1.000	0.931	-1.010	0.644	-1.010	0.548
GTF2I	TC07000458.hg.1	-1.140	0.003	1.030	0.837	1.050	0.611	1.020	0.508
HMOX1	TC22000259.hg.1	1.120	0.003	-1.090	0.017	-1.090	0.047	-1.000	0.865
HSPA1A	TC06001547.hg.1	1.230	0.386	1.030	0.903	-1.020	0.758	-1.060	0.877
HSPA1A	TC6_apd_hap1000088.hg.1	1.160	0.465	1.030	0.722	-1.020	0.585	-1.050	0.930
HSPA1A	TC6_cox_hap2000174.hg.1	1.160	0.418	1.020	0.622	-1.020	0.596	-1.050	0.940
HSPA1A	TC6_dbb_hap3000163.hg.1	1.210	0.414	1.020	0.784	-1.010	0.636	-1.030	0.877
HSPA1A	TC6_qbl_hap6000164.hg.1	1.130	0.424	1.010	0.767	-1.010	0.843	-1.020	0.864
HSPA1A; HSPA1B	TC06000384.hg.1	2.860	0.040	-1.010	0.993	-1.120	0.682	-1.110	0.549
HSPA1A; HSPA1B	TC6_apd_hap1000039.hg.1	2.920	0.038	-1.050	0.869	-1.150	0.552	-1.100	0.577
HSPA1A; HSPA1B	TC6_cox_hap2000078.hg.1	2.920	0.039	-1.050	0.873	-1.150	0.556	-1.100	0.578
HSPA1A; HSPA1B	TC6_dbb_hap3000069.hg.1	2.840	0.037	-1.040	0.841	-1.150	0.523	-1.100	0.586
HSPA1A; HSPA1B	TC6_qbl_hap6000069.hg.1	2.930	0.038	-1.060	0.862	-1.160	0.546	-1.090	0.583
HSPA1B; HSPA1A	TC06000385.hg.1	3.450	0.033	1.010	0.958	-1.140	0.723	-1.160	0.538
HSPA1B; HSPA1A	TC6_apd_hap1000040.hg.1	3.900	0.030	1.020	0.918	-1.140	0.757	-1.150	0.523
HSPA1B; HSPA1A	TC6_cox_hap2000079.hg.1	3.700	0.031	1.020	0.908	-1.140	0.757	-1.160	0.511

HSPA1B; HSPA1A	TC6_dbb_hap3000070.hg.1	3.830	0.031	1.010	0.917	-1.140	0.758	-1.160	0.523
HSPA1B; HSPA1A	TC6_qbl_hap6000070.hg.1	3.690	0.029	1.020	0.922	-1.130	0.756	-1.150	0.528
KRT1	TC12001532.hg.1	-1.420	0.001	-1.080	0.244	-1.110	0.057	-1.020	0.267
LOC100129518; SOD2	TC06004142.hg.1	-1.010	0.373	1.020	0.512	1.030	0.702	1.010	0.815
LPO	TC17000712.hg.1	1.090	0.003	-1.050	0.179	-1.010	0.262	1.040	0.557
MB	TC22000697.hg.1	1.130	0.002	-1.060	0.129	-1.090	0.009	-1.030	0.216
MB; LOC284912	TC22000698.hg.1	1.180	0.094	-1.040	0.818	-1.030	0.650	1.010	0.657
MBL2	TC10001301.hg.1	1.130	0.006	-1.010	0.446	-1.050	0.076	-1.050	0.550
MGST3	TC01001444.hg.1	1.100	0.028	1.010	0.913	1.030	0.273	1.020	0.183
MGST3; LOC100505828	TC01001443.hg.1	1.040	0.418	-1.030	0.388	-1.050	0.199	-1.020	0.482
MPO	TC17001727.hg.1	1.120	0.002	-1.070	0.048	-1.070	0.004	-1.000	0.676
MPV17	TC02001681.hg.1	-1.070	0.003	-1.040	0.118	-1.060	0.026	-1.020	0.474
MSRA	TC08000071.hg.1	1.090	0.005	-1.040	0.200	-1.060	0.062	-1.020	0.477
MSRA	TC08000073.hg.1	1.110	0.007	-1.020	0.223	-1.070	0.088	-1.040	0.774
MT3	TC16000465.hg.1	1.050	0.053	-1.050	0.524	-1.010	0.283	1.040	0.103
NCF1	TC07000459.hg.1	1.060	0.009	-1.040	0.032	-1.050	0.064	-1.010	0.682
NCF2	TC01003616.hg.1	1.020	0.539	-1.030	0.326	-1.020	0.317	1.010	0.844
NCF4	TC22000270.hg.1	1.100	0.063	-1.030	0.520	-1.030	0.993	1.000	0.443
NOS2	TC17002894.hg.1	1.100	0.006	-1.030	0.206	-1.040	0.199	-1.010	0.983
NOX4	TC11002178.hg.1	1.020	0.229	1.010	0.892	1.000	0.800	-1.010	0.916
NOX5; SPESP1	TC15000630.hg.1	1.070	0.000	-1.030	0.039	-1.030	0.231	-1.000	0.507
NQO1	TC16001225.hg.1	-1.140	0.042	1.140	0.027	1.040	0.870	-1.100	0.249
NUDT1	TC07000029.hg.1	1.030	0.420	-1.030	0.601	-1.000	0.961	1.030	0.531
OXR1; RP11-395G23.3	TC08000660.hg.1	1.030	0.026	1.060	0.020	1.050	0.061	-1.000	0.933
OXSR1	TC03000188.hg.1	1.040	0.155	1.110	0.008	1.060	0.040	-1.040	0.228
PDLIM1	TC10001549.hg.1	-1.020	0.210	1.020	0.247	1.000	0.486	-1.020	0.518
PNKP	TC19001727.hg.1	1.060	0.010	-1.040	0.071	-1.000	0.877	1.040	0.072
PRDX1	TC01002611.hg.1	1.020	0.594	1.030	0.077	-1.030	0.554	-1.050	0.042
PRDX2	TC19001210.hg.1	-1.080	0.245	-1.020	0.491	-1.090	0.046	-1.080	0.060
PRDX3	TC10001702.hg.1	-1.170	0.017	1.050	0.325	-1.050	0.274	-1.100	0.068

PRDX4	TC0X000110.hg.1	-1.240	0.002	1.080	0.165	1.030	0.519	-1.060	0.341
PRDX5	TC11000597.hg.1	1.010	0.846	-1.020	0.896	-1.050	0.752	-1.030	0.792
PRDX6	TC01001510.hg.1	-1.020	0.565	1.040	0.083	1.010	0.565	-1.030	0.230
PREX1	TC20000920.hg.1	1.140	0.025	-1.030	0.644	1.010	0.716	1.040	0.396
PRNP	TC20000051.hg.1	-1.240	0.002	-1.010	0.781	-1.050	0.128	-1.030	0.093
PTGS1	TC09000614.hg.1	1.090	0.002	-1.030	0.337	-1.030	0.248	-1.000	0.870
PTGS2	TC01003638.hg.1	1.070	0.010	-1.000	0.914	1.000	0.441	1.010	0.596
PXDN	TC02001512.hg.1	1.040	0.117	-1.020	0.212	-1.010	0.274	1.000	0.626
RNF7	TC03000764.hg.1	-1.130	0.033	1.080	0.128	-1.010	0.690	-1.090	0.074
SCARA3	TC08000209.hg.1	1.060	0.058	-1.000	0.891	-1.050	0.148	-1.040	0.212
SCRT2; SRXN1; PS1TP5; RP5-850E9.3	TC20000544.hg.1	1.030	0.132	-1.040	0.101	-1.050	0.322	-1.010	0.508
SEPP1	TC05001310.hg.1	1.010	0.167	-1.010	0.568	-1.000	0.313	1.010	0.326
SFTPD	TC10001458.hg.1	1.120	0.020	-1.070	0.105	-1.040	0.411	1.030	0.351
SIRT2	TC19001508.hg.1	1.010	0.266	-1.040	0.031	-1.040	0.019	-1.000	0.784
SOD1	TC21000111.hg.1	-1.030	0.419	1.130	0.019	1.050	0.172	-1.080	0.143
SOD2; LOC100129518	TC06004141.hg.1	1.020	0.362	1.070	0.012	1.030	0.274	-1.040	0.250
SOD3	TC04000173.hg.1	-1.010	0.984	-1.020	0.509	1.030	0.610	1.060	0.297
SQSTM1	TC05001053.hg.1	1.000	0.564	1.020	0.668	-1.010	0.293	-1.030	0.593
STK25	TC02002951.hg.1	1.020	0.110	-1.030	0.067	1.010	0.702	1.030	0.117
TPO	TC02000013.hg.1	-1.050	0.019	-1.040	0.014	-1.060	0.003	-1.020	0.719
TTN	TC02005047.hg.1	1.050	0.011	-1.030	0.744	1.020	0.067	1.040	0.114
TXN	TC09001473.hg.1	-1.040	0.504	1.080	0.066	1.020	0.820	-1.060	0.056
TXNRD1	TC12000810.hg.1	-1.030	0.665	1.100	0.036	1.020	0.432	-1.080	0.154
TXNRD2	TC22000502.hg.1	1.030	0.138	-1.040	0.025	-1.060	0.014	-1.010	0.634
UCP2	TC11002074.hg.1	1.160	0.016	-1.030	0.510	1.010	0.806	1.040	0.360
VIMP	TC15002006.hg.1	-1.090	0.035	1.100	0.008	1.090	0.186	-1.020	0.209

Supplementary Table 6: List of genes of the four groups of gravity-sensitive transcripts in figure 1 a-d. If genes are represented by more than one transcript in the group, the gene name is listed only once.

**hypergravity-sensitive transcripts**		**hypergravity-sensitive transcripts, 1g IF-controlled**		**microgravity-sensitive transcripts, BL-controlled**		**microgravity-sensitive transcripts, BL and 1g IF-controlled**	
**up-regulated**	**down-regulated**	**up-regulated**	**down-regulated**	**up-regulated**	**down-regulated**	**up-regulated**	**down-regulated**
DUOX2	CAT	EPHX2	CYBA	CYBA	PXDN	CYBA	PXDN
EPHX2	CYBB	GPX5	CYBB	PTGS1			
GPX5	FOXM1	GPX6	FOXM1				
GPX6	GCLC	GPX7	GCLM				
GPX7	GSR	KRT1	GSR				
GPX7	GSS	LOC653361	GTF2I				
HMOX1	GSTZ1	NCF1	NCF4				
HSPA1A	GTF2I	NOS2A	SOD2				
KRT1	MPV17	PTGS2	SQSTM1				
NOS2A	NCF4	PXDN	TXNRD2				
PXDN	TXNRD2	SCARA3					
SCARA3		SOD3					
SOD2		TPO					
SOD3		TTN					
TPO							
TTN							

Supplementary Table 7: List of genes of the two groups of gravity-sensitive transcripts in figure 2 a and b. If genes are represented by more than one transcript in the group, the gene name is listed only once.

**hyperravity-sensitive transcripts**		**microgravity-sensitive transcripts, BL-controlled**	
up-regulated	down-regulated	up-regulated	down-regulated
**CAT**	ALOX12	ALOX12	GCLM
**CYBB**	GPX5	GPX7	GSR
**GCLC**	KRT1	LOC653361	MSRA
**GCLM**	MB	MT3	OXSR1
**GPX4**	MT3	NCF1	PRDX4
**GSR**	PNKP	PXDN	PRNP
**GTF2I**		SOD3	PTGS2
**HSPA1A**			SELS
**MSRA**			SOD1
**NQO1**			SOD2
**OXR1**			
**OXSR1**			
**PRDX3**			
**PRDX4**			
**PREX1**			
**PRNP**			
**PTGS1**			
**PTGS2**			

**SELS**			
**SOD1**			
**SOD2**			
**TXNRD1**			

Supplementary Table 8: Lists of genes in the four reactions categories shown in figure 4. Human myelomonocytic U937 cells were exposed to 20s 1.8g and 75s max. 13.5g of hypergravity, gene regulation of 188 transcripts involved in oxidative stress response were monitored over these 2 time points. If genes are represented by more than one transcript in the group, the gene name is listed only once.

**continuous response**	**adaption**	**late response**	**no response**
PTGS2	CYBA	ALOX12	ALB
	CYBB	CAT	ALOX12
	EPHX2	GCLC	AOX1
	FOXM1	GCLM	APOE
	FOXM1	GPX4	ATOX1
	GCLM	GTF2I	BNIP3
	GPX5	MB	CCL5
	GPX6	MSRA	CCS
	GPX7	MT3	CYGB
	GSR	NQO1	DHCR24
	GTF2I	OXR1	DUOX1
	KRT1	OXSR1	DUOX2
	KRT1	PNKP	DUSP1
	LOC653361	PRDX3	EPX
	NCF1	PRDX4	FTH1
	NCF4	PREX1	GPX1
	NOS2A	PRNP	GPX2
	PXDN	PTGS2	GPX3
	PXDN	SELS	GPX4
	SCARA3	SOD1	GPX5

	SOD2	TXNRD1	GPX7
	SOD3		GSS
	SQSTM1		GSTP1
	TPO		HMOX1
	TTN		LPO
	TXNRD2		MB
			MBL2
			MGST3
			MPO
			MPV17
			MT3
			NCF2
			NOX4
			NOX5
			NQO1
			NUDT1
			OXR1
			PDLIM1
			PRDX1
			PRDX2
			PRDX5
			PRDX6
			PTGS2
			PXDN
			RNF7
			SCARA3
			SELS
			SEPP1
			SFTPD
			SIRT2

			SOD1
			SOD2
			SOD3
			SQSTM1
			SRXN1
			STK25
			TPO
			TTN
			TXN
			TXNRD2
			UCP2

Supplementary Table 9:. Lists of genes in the four reactions categories shown in figure 5. Human myelomonocytic U937 cells were exposed to 20 s and 300s of microgravity, gene regulation of 188 transcripts involved in oxidative stress response were monitored over these 2 time points. If genes are represented by more than one transcript in the group, the gene name is listed only once.

continuous response	adaption	late response	no response
	CYBA	ALOX12	ALB
	PXDN	GCLM	ALOX12
		GPX7	AOX1
		GSR	APOE
		LOC653361	ATOX1
		MSRA	BNIP3
		MT3	CAT
		NCF1	CCL5
		OXSR1	CCS
		PRDX4	CYBA
		PRNP	CYBB
		PTGS2	DHCR24
		PXDN	DUOX1
		SELS	DUOX2
		SOD1	DUSP1
		SOD3	EPHX2
			EPX
			FOXM1
			FTH1
			GCLC

			GCLM
			GPX1
			GPX2
			GPX3
			GPX4
			GPX5
			GPX6
			GPX7
			GSS
			GSTP1
			GTF2I
			HMOX1
			KRT1
			LPO
			MB
			MBL2
			MGST3
			MPO
			MPV17
			MT3
			NCF2
			NCF4
			NOS2A
			NOX4
			NOX5
			NQO1
			NUDT1
			OXR1
			OXSR1
			PDLIM1

			PNKP
			PRDX1
			PRDX2
			PRDX3
			PRDX5
			PRDX6
			PREX1
			PTGS2
			PXDN
			RNF7
			SCARA3
			SELS
			SEPP1
			SFTPD
			SIRT2
			SOD1
			SOD2
			SOD3
			SQSTM1
			SRXN1
			STK25
			STK25
			TPO
			TTN
			TXN
			TXNRD1
			TXNRD2
			UCP2
